# Supplementary material for: Flavonoid Biosynthesis Genes in Triticum aestivum L.: Methylation Patterns in Cis-Regulatory Regions of the Duplicated CHI and F3H Genes
Source: Biomolecules. 2022 May 11;12(5):689. doi: 10.3390/biom12050689 (PMC9138379; doi:10.3390/biom12050689)
Supplement: Supplementary file 1 [file biomolecules-12-00689-s001.zip › biomolecules-1628042-supplementary.pdf]

Supplementary Material

1. Section S1:The alignment of *TaCHI* promoters

```
          1                                                                                                 100
TaCHI-A1  AAAAGAATTGCCCAGTTAAGCACAAGATAATTCCAAAAGGTTGTGATTTTACTCGTAGTACAAACAATTGGAGCTTTTCCACACCCTACAAGGTGATTTC
TaCHI-D1                                                                       GGAGCTTTTCCACACCCTATAAGGTGGTTTC
TaCHI-B1                                                                            CGGACCCAAACGGACAAAAAGCGAAC
          101                                                                                               200
TaCHI-A1  TTTAAT---------ATCGGTCTGCAGTGCACGGTGCAGTTTTAGTT-TTATATTCTTTTTTGAAACATGCAGTTTTAGTTTGGTGATATGTTGAGTTCC
TaCHI-D1  TTTAATTCTGTTGAGATCGGTCTGCAGTGCACGGTGCAGTTTTTGTT-TT----TGTTTTTTGAAACATTCAGTTTTAGTTTGGTGATATGTTGAGTTCC
TaCHI-B1  AAAAT----------CACCGTCCGTTTGGGTCGGCACCGTTGGAGTTGCTCTAATCCTTATTAATTCATGCC---TGAGGTAGGCGGAACATTA----CT
          201                                                                                               300
TaCHI-A1  TTTCCTTTGCCAGACCGGCAGACCCTGCATCGGCCAACA-GCACGAGCGTCTCGTCTGGGCTCCTGCGTGAGTT-----------------GAATGGTA-
TaCHI-D1  TTTGCTTTACCAGACCGGCAGACCCTGCATCGGCCAGCA-ACACGAGTGTCTGGTCTGGACTCGTGCGTGAGTTTTTTTTTTTTTTTGAGGGACTCGTGC
TaCHI-B1  CTTGCCTACCCCGTAGGGTGGTTTCTTTTGAGATCGGTGTGCTTTGGTGACATGT-TGAGCCGTTGTGTCTGGTGT--------------GGGCTCGTGC
          301                                                                                               400
TaCHI-A1  -----------GCTCGCTTGACCTCGCCGCAAAACCTGGCGCGCGGCCACGTCCGT----------CCTCTCAACGTGCCTCTCCTAACTACCACTAACC
TaCHI-D1  GTGAGTTGAATGGTAGCTTGACCTCGCCGCAAAACCTGGCGCGCGGACACGTCCTCTCTCT----GCCCCTCAACGTGCCTCTCCTAACTACCACTAACC
TaCHI-B1  GTGAGTTGAATGGTAGCTTGACGTCGCCGCAAAACCTGGCGCGCGGCCACGTCCGTCCTCTCTTTGCCCCTCAACGTGCCTCTCCCAACTACCACTAACC
          401                                                                                               500
TaCHI-A1  ACGCCCCCAGACGCGCCCACAAACTACATCTTTGCCACAAGGCACA-AGTTCACCCCCCGTCTACG--------------CGTAGGCCAAAGAACTAGAC
TaCHI-D1  ACGCCCCCAGATGCGCCCACAAACTACATCTCTGCCACAAGGACAA-GGTCCACCCCCCGTCACCGTCTAGTCTAGGCCGCGTAGGCCAGAGAACCAGAC
TaCHI-B1  ACGCCCCCATATGCGCACACAAACTACATCTTTGCCACAAGGCACAAGGTCCACCCTCCGTTACCGTCTAGTCTAGGCCGCGTAGGCCAGAGAACCAGAC
          501                                                                                               600
TaCHI-A1  CAGATTACGGCCGTGGCATAATCGCAATTACGGGCAGAGTCCACGGGCGTTTATATAGCCGCTGGGGTGTCATATAGGAGAGG---GCACCACCACCCCA
TaCHI-D1  CAGATTACGGCCGTGGCATAATCGCAATTACGGGCAGAGTCGACGGGCGTTTATATAGCTGTTGCGGTGTCATATAGGGGGGGCACGCACCACCACCCCA
TaCHI-B1  CAGATTACAGCCGTGGCACAATCGCAATTACGGGCAGAGTGTACGGGCGTTTATATAGCTGTTGCGGTGTCATATAGGGGGGGCACGCACCACCACCCCA
          601                                                                                               700
TaCHI-A1  GCTGACCCATCCCGTGCGCCCACGCCTCAACAGATCGCACCTACCCTGCCGGGCCGGCCTGCCCCCATCACATCAATACAAGCGTTCGCTCGCTACGTGCC
TaCHI-D1  GCTACCCATCCCGTGCGCCTACGCCTCGCCAGATCGCACCTACCCTGCCGG-----CCTGCCCCCATCACATCAATACAAGTGTTCGCTCGCTACGTGCC
TaCHI-B1  GCTACCCATCCCGTGCGCCCACGCCTCGCCAGATCGCACCTACCCTGCCGC-----CCTGCCCCCGTCCCATCAATACAAGTGTTTGCTCGCTACGTGCC
          701                                        747
TaCHI-A1  GTC----GTCTCCGGAGACCCTCCGCGCA-CCA-CCG-----AGACC
TaCHI-D1  GTC----GTCTCCGGAGACCCTCCGTGCA-CCA-CC
TaCHI-B1  GTCCGTCGTCTCCGGAGACCCTCCATCCATCCATCCGGCGATATACC
```

2. Section S2:The alignment of *TaF3H* promoters

```
          1                                                                                                 100
TaF3H-B1  TCTACAAGCCATGGAAATATGACGTGTCATGATGTATAGGTTTTAGATATTGGGCGAACCAATCTGTGGTTGGATGGTTAAAGGGACTCTGATATCCCCA
TaF3H-B2                                           ATTTGCAACCGAGCGAGC--TTAGATGGTTAGATATTTCTTG------TGGTGGAACTA
          101                                                                                               200
TaF3H-B1  GTCCACTAAGGTTCAAA--TCTGGTGCTTGAGTTTATTCC-TGGATTATTTCAGGA-TTTTCGACGATGTGCATTCAGTAGGAGAAGACGTTCCCGTCGA
TaF3H-B2  GCCCATCATGGTTCATAGTCCTAAGGCCTTATTTGGTTTGGAGGAATTTCATAGGACTTTTATACGATAGGATTCTTATAGGAAATTT--TTCTTAAAGC
          201                                                                                               300
TaF3H-B1  CAACGAGTTGCCCACGATGACTTCGTAAATTTCAAGATAATATGCCGATTCGGTCTTTCGGAGGTGCTCATAGGGGTAGGGTGTGCGTGTGTGCGTTTAT
TaF3H-B2  CTATTAATTCATAGGAATGAATT--TTTATTCCTACATAGAAT-TGGTTCCTATCCTTCA------CACTTTAAAGTAAATAAA----TATGAATCTAG
          301                                                                                               400
TaF3H-B1  AGGGATGAGTGTATGCACGTATATATGAGCGCTTGTATCTGGACTGTGTTAAAAAAAAGTTCTAGATCTTGGTTGGTGTATAGCGGGTGTCCAGTGGTGT
TaF3H-B2  ACTCACTGGAAAAAAATCATAT-TATG------TGAAACTCATAGGATTTGAGAGACTTGTC---ATCTCATTTCCTA-----CAAGTTTTCTATT---T
          401                                                                                               500
TaF3H-B1  GTGTGCTTGTGATGTGTGTGTTGTGCGTATATGGATCGATCGATACGGCTGGATGGATGCATGGGTGCGGCCCGGTTAGTTGCGCACCTAACACGAGTGC
TaF3H-B2  CTATGATAATGGTATAT--------CGTAT--GAACCAAAAGAAGC--CTAATTTGACACTCGTGCATG---CATTTTTCTGAATTTATTTCA-------
          501                                                                                               600
TaF3H-B1  CCGAGGGCGACTTCCTTGGTTAGTTGCGTGCGCACGCGCGACTTGCAGGTCGCGTCTGCCCCTCATCTTCCAACGTAGTATACACAATGCCCTCTTCCCG
TaF3H-B2  --------GACTTTCT-GGTGATGTTCATTCA-GTGCGAGACAT-----------CT--CCCTAATTTAAAGATGTTGTG---CCGGTTCAGTATTTC--
          601                                                                                               700
TaF3H-B1  GCCGTATATATCCGCGTGCGTACAACGTCCAGAAAACCACAACCCAAACGGCCGAGCCTTTCATCAAGGAACAAACCATCATACGAGACAGACGCCGGCC
TaF3H-B2  -----AAAGGTACTCAGGCGTTCATAGGC---ATCTCCCTAATTTAAATATATGTACCCTCCTTTCC---ATCTATAATCATACGAGACAGACGTA--CA
          701                           735
TaF3H-B1  GCGACACAAGCGGACGATCCCCGGAGTGCGGATCG
TaF3H-B2  GATCGATAACTGGTAGATAGCG
```

3. Section S3:CpG island prediction in promoters of *TaCHI* and *TaF3H* genes made with MethPrimer 2.0

Sequence Name: **Triticum aestivum chalcone flavanone isomerase (Chi-A1)**

Sequence Length: 1652 bp

Promoter Sequence: 1-754 bp

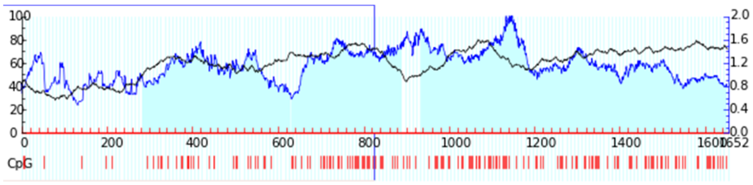

CpG Island Prediction

No.	Island size	Island start	Island end	GC% percent	O/E ratio
Island 1	346	284	629	50.0	0.6
Island 2	258	632	889	50.0	0.6

Sequence Name: **Triticum aestivum chalcone flavanone isomerase (Chi-B1)**

Sequence Length: 1550 bp

Promoter Sequence: 1-636 bp

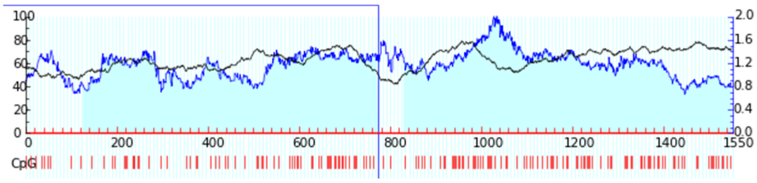

CpG Island Prediction

No.	Island size	Island start	Island end	GC% percent	O/E ratio
Island 1	645	128	772	50.0	0.6
Island 2	719	831	1549	50.0	0.6

Sequence Name: **Triticum aestivum chalcone flavanone isomerase (Chi-D1)**

Sequence Length: 1559 bp

Promoter Sequence: 1-645 bp

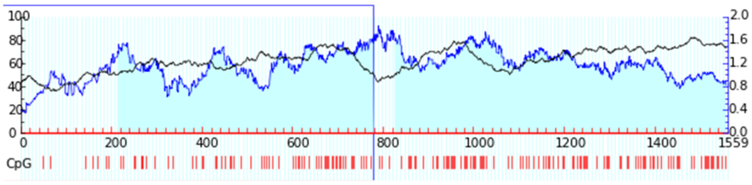

CpG Island Prediction

No.	Island size	Island start	Island end	GC% percent	O/E ratio
Island 1	564	215	778	50.0	0.6
Island 2	733	826	1558	50.0	0.6

Sequence Name: **Triticum aestivum flavanone 3-hydroxylase (F3H-B1)**

Sequence Length: 2811 bp

Promoter Sequence: 1-731 bp

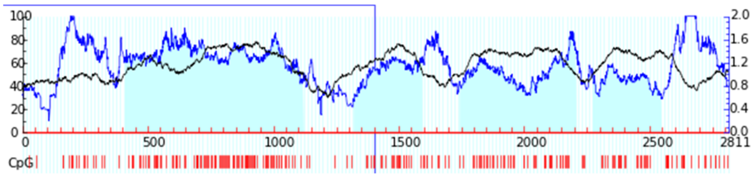

CpG Island Prediction

No.	Island size	Island start	Island end	GC% percent	O/E ratio
Island 1	711	409	1119	50.0	0.6
Island 2	271	1324	1594	50.0	0.6
Island 3	462	1746	2207	50.0	0.6
Island 4	273	2273	2545	50.0	0.6

Sequence Name: **Triticum aestivum flavanone 3-hydroxylase (F3H-B2)**

Sequence Length: 1989 bp

Promoter Sequence: 1-577 bp

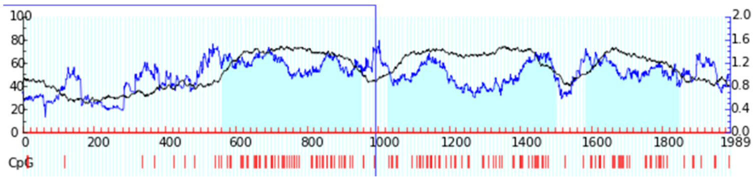

CpG Island Prediction

No.	Island size	Island start	Island end	GC% percent	O/E ratio
Island 1	393	562	954	50.0	0.6
Island 2	474	1029	1502	50.0	0.6
Island 3	264	1586	1849	50.0	0.6

4. Section S4:The alignment of *TaCHI* promoter sequences and assembled promoter sequences after bisulfite treatment

Coleoptiles *TaChi-A1*

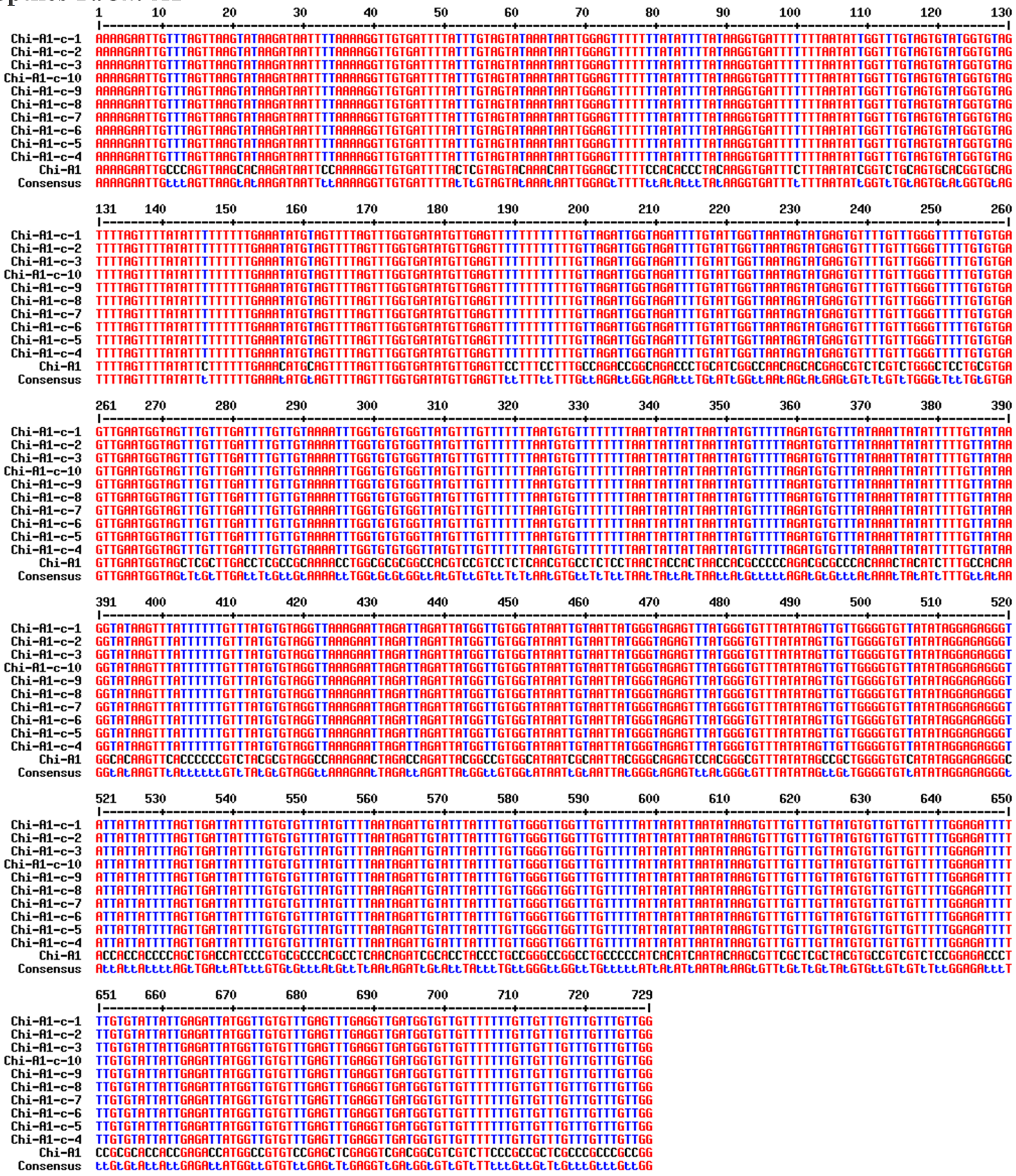

Roots *TaChi-A1*

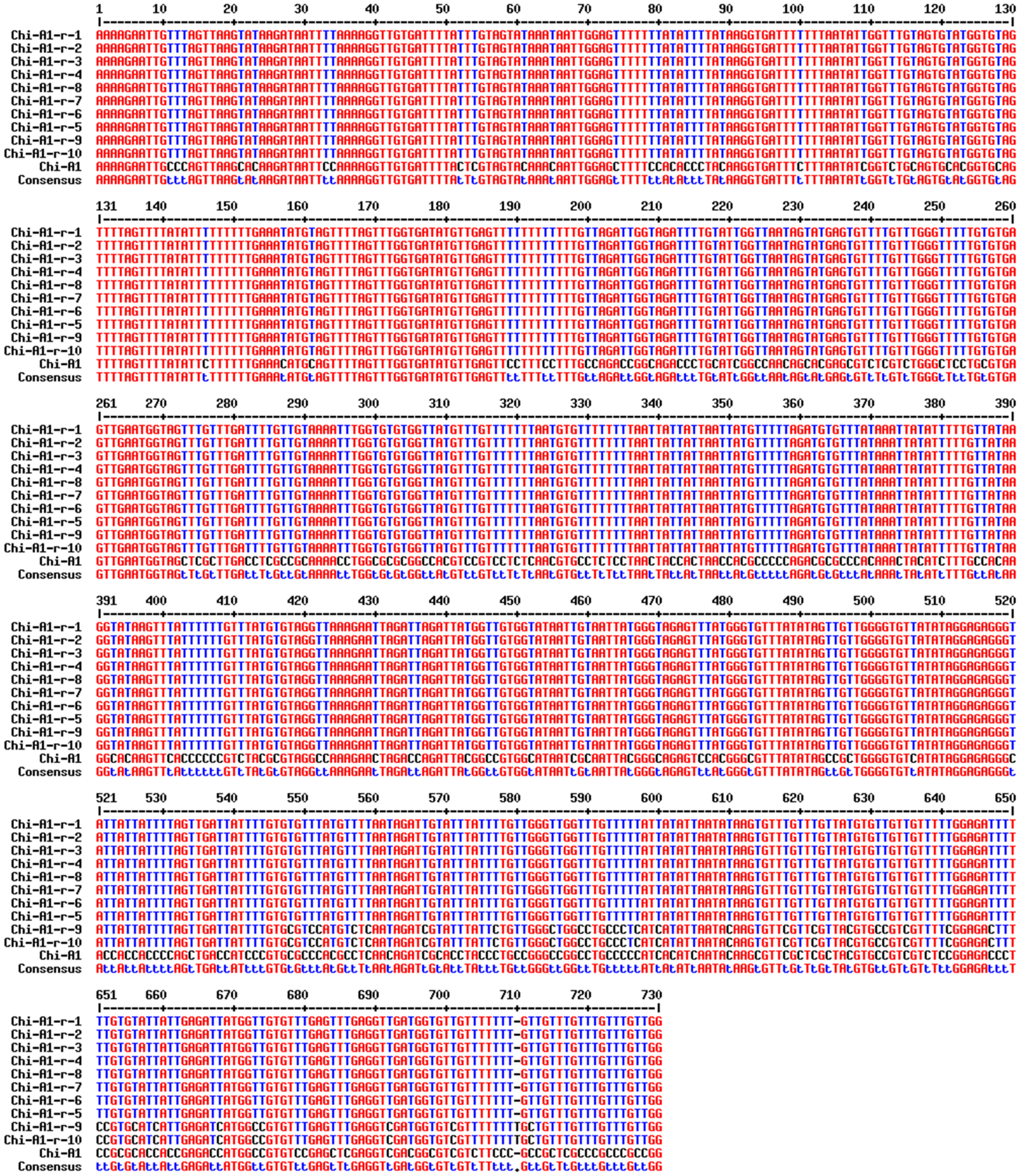

Coleoptiles *TaChi-B1*

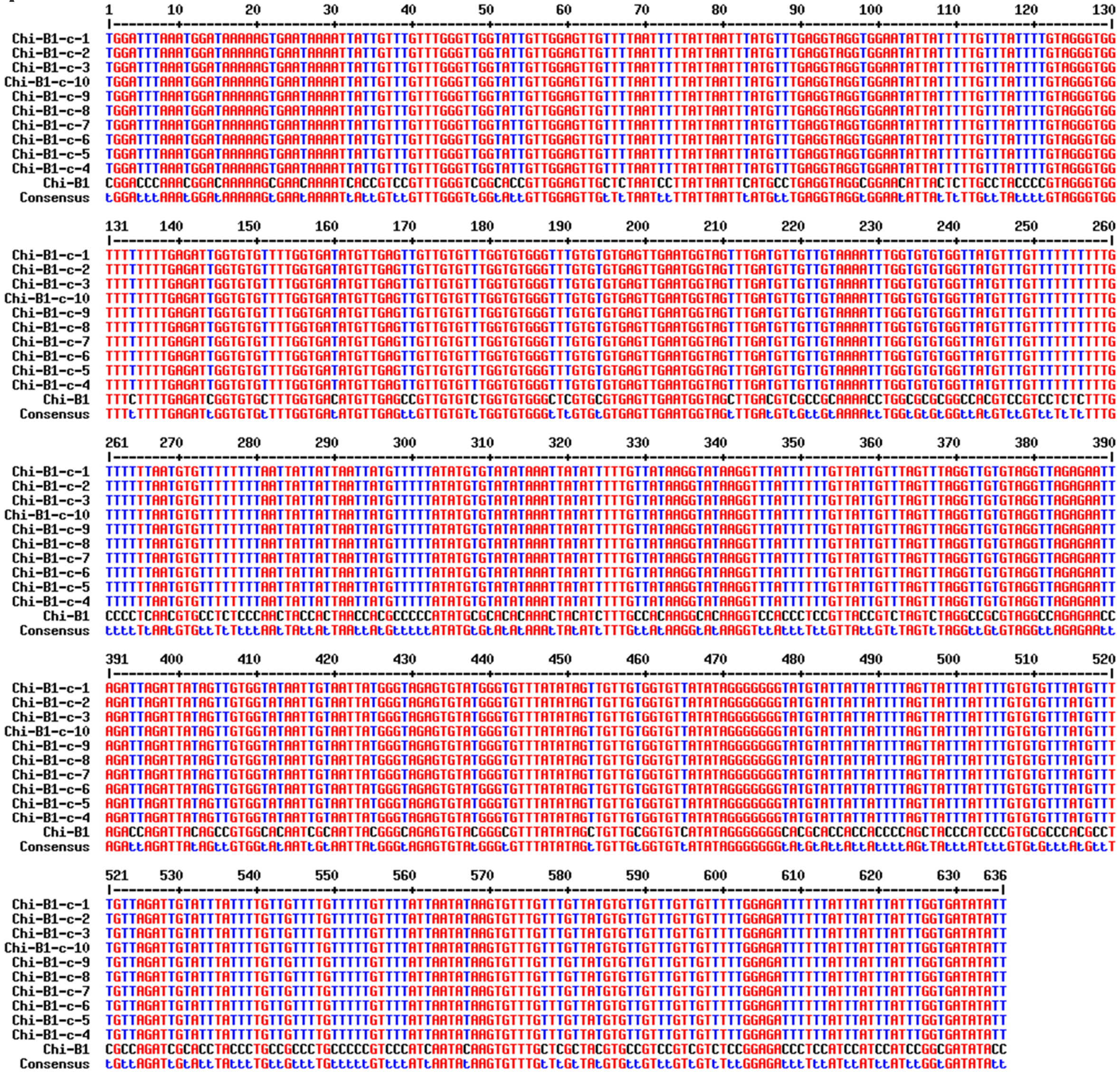

Roots *TaChi-B1*

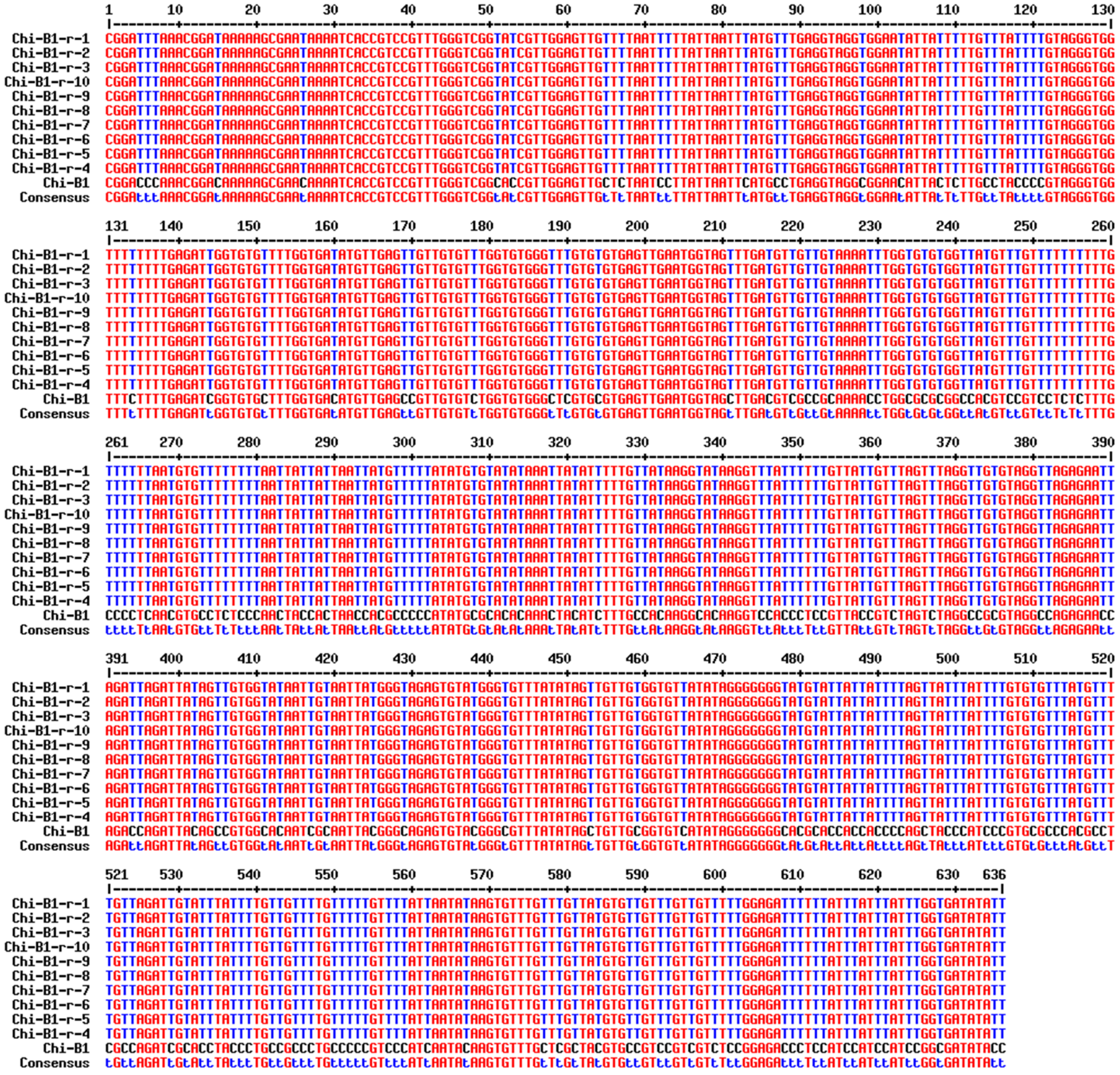

Coleoptiles *TaChi-D1*

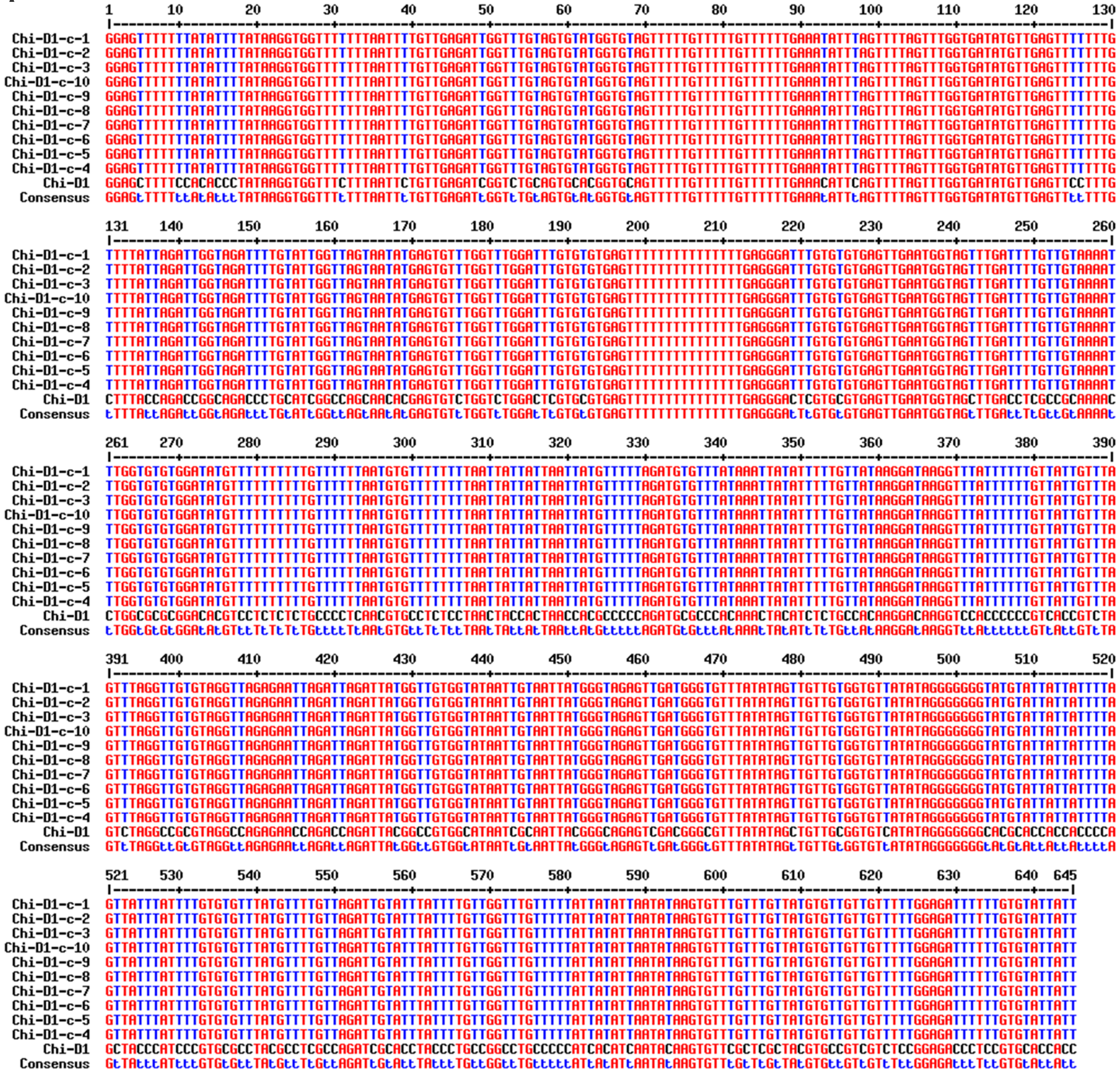

Roots *TaChi-D1*

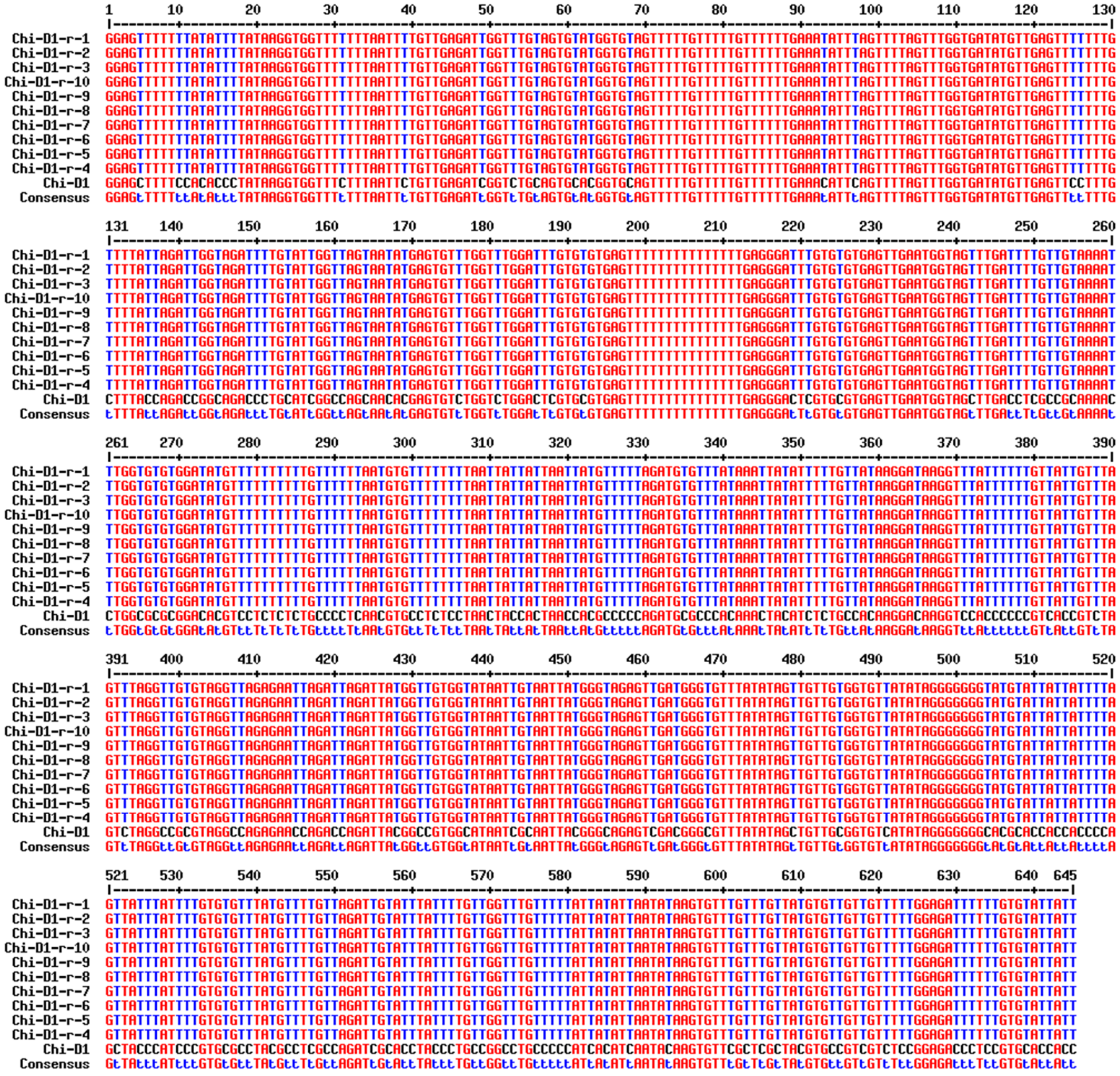

Coleoptiles *TaF3H-B1*

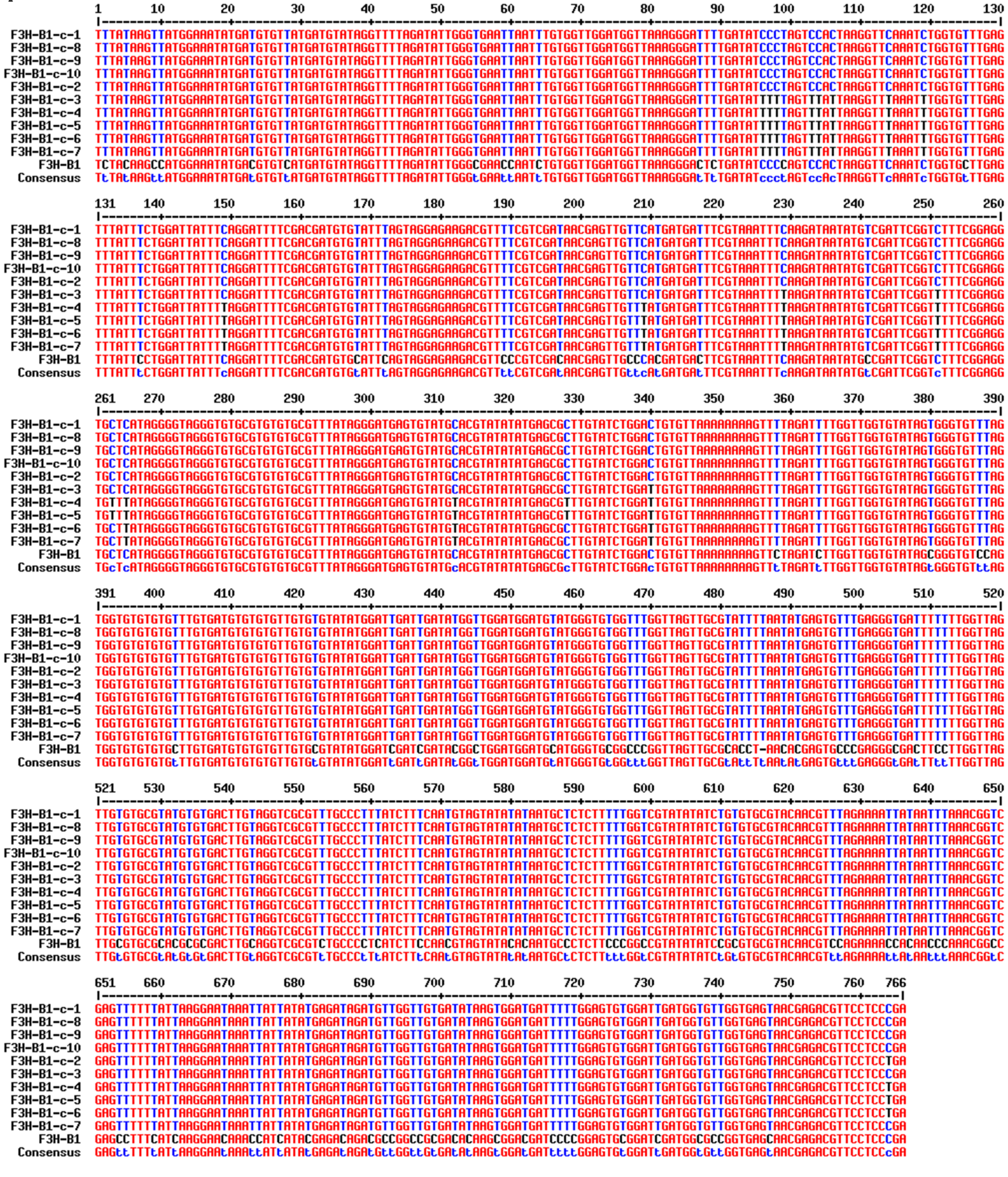

Roots *TaF3H-B1*

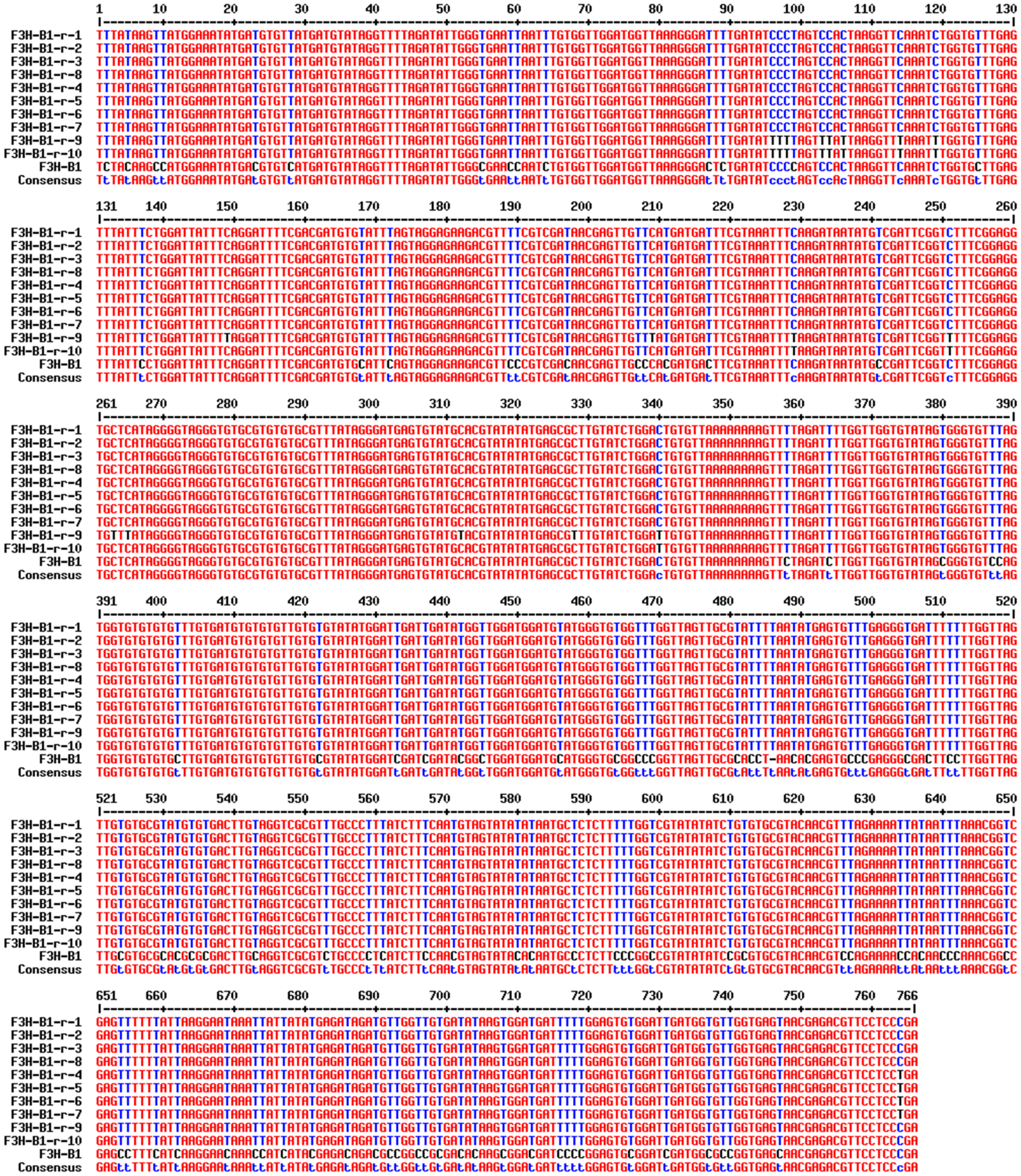

Coleoptiles *TaF3H-B2*

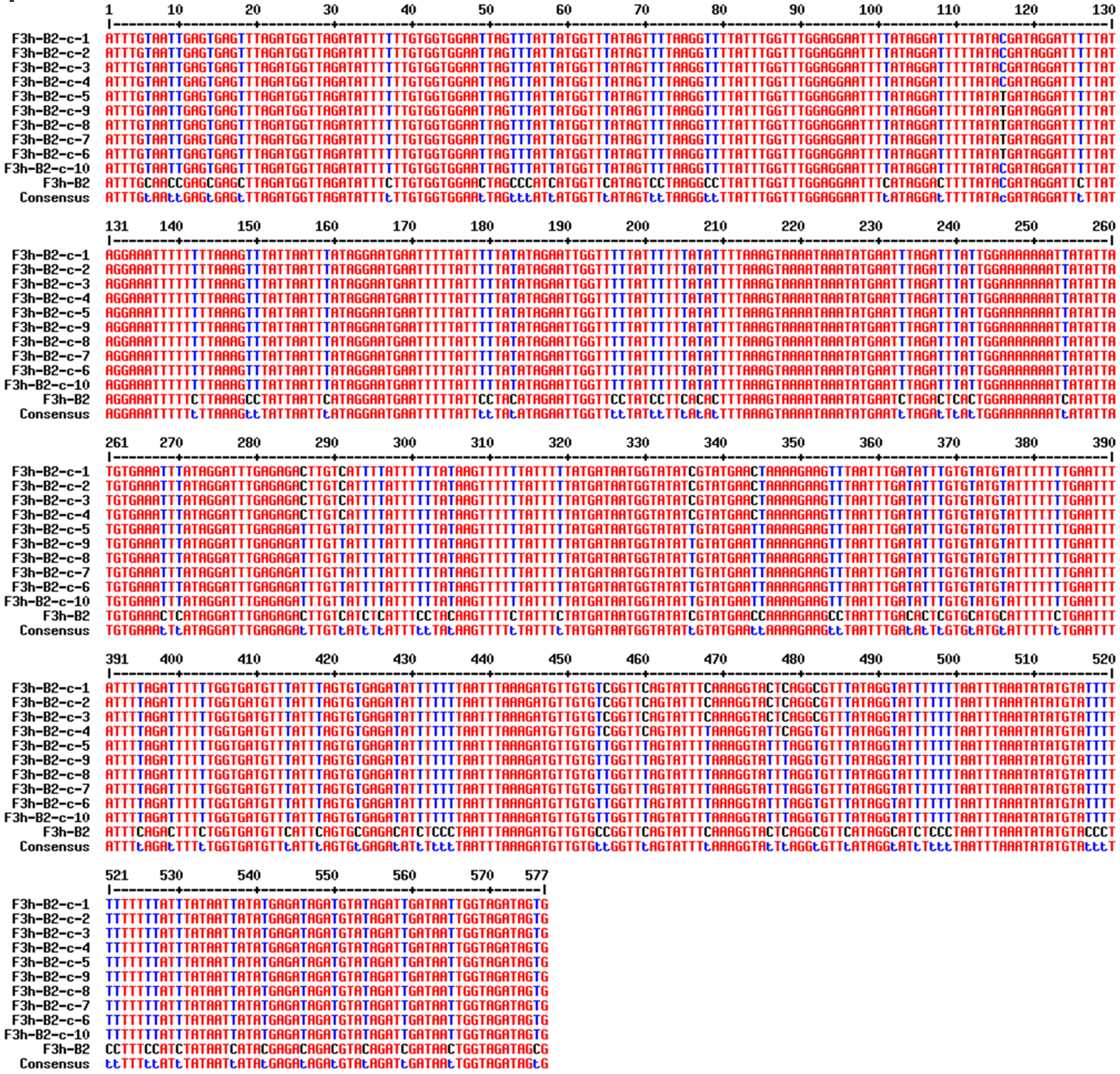

Roots *TaF3H-B2*

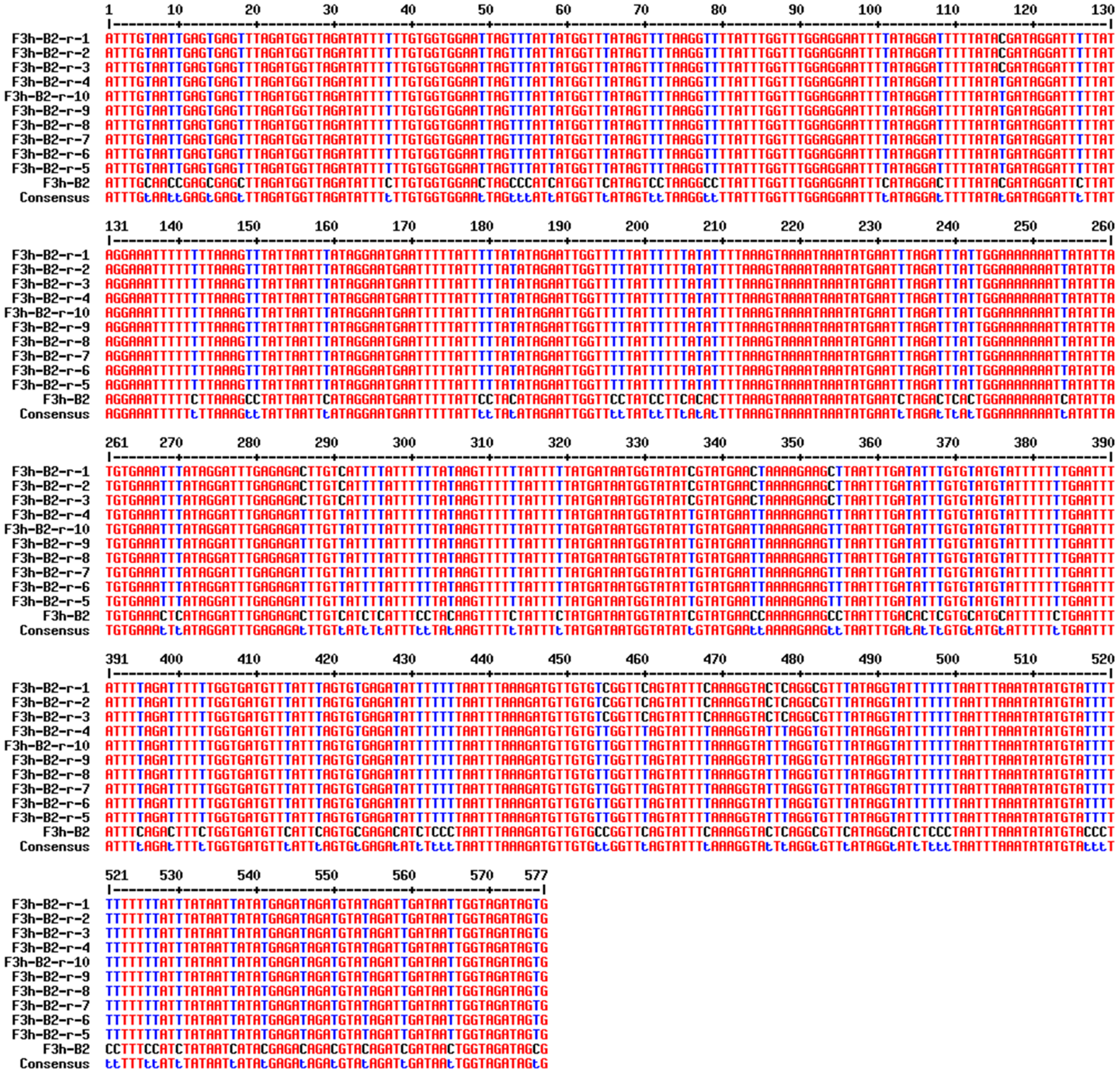

5. Section S5:Results of the signal scan search request in promoters of *TaCHI* and *TaF3H* genes made with New PLACE and PlantPAN 3.0

The diagrams show the arrangement of elements in the promoter, the tables contain a description of the elements. Elements in the tables are highlighted in colors: light-dependent activation (yellow), TF-dependent elements (red), tissue- specific elements (green), stress-specific elements (blue). Sequences that correspond to methylation labels are highlighted in bold.

(+) = Current Strand
(-) = Opposite Strand

>TaChi-A1
AAAAGAATTGCCCAGTTAAGCACAAGATAATTCCAAAAGGTTGTGATTTTACTCGTAGTACAAACAATTGGAGCTTTTCCA
CACCCTACAAGGTGATTTCTTTAATATCGGTCTGCAGTGCACGGTGCAGTTTTAGTTTTATATTCTTTTTTGAAACATGCA
GTTTTAGTTTGGTGATATGTTGAGTTCCTTTCCTTTGCCAGACCGGCAGACCCTGCATCGGCCAACAGCACGAGCGTCTCG
TCTGGGCTCCTGCGTGAGTTGAATGGTAGCTCGCTTGACCTCGCCGCAAAACCTGGCGCGCGGCCACGTCCGTCCTCTCAA
CGTGCCTCTCCTAACTACCACTAACCACGCCCCCAGACGCGCCCACAAACTACATCTTTGCCACAAGGCACAAGTTCACCC
CCCGTCTACGCGTAGGCCAAAGAACTAGACCAGATTACGGCCGTGGCATAATCGCAATTACGGGCAGAGTCCACGGGCGTT
TATATAGCCGCTGGGGTGTCATATAGGAGAGGGCACCACCACCCCAGCTGACCATCCCGTGCGCCCACGCCTCAACAGATC
GCACCTACCCTGCCGGGCCGGCCTGCCCCCATCACATCAATACAAGCGTTCGCTCGCTACGTGCCGTCGTCTCCGGAGACC
CTCCGCGCACCACCGAGACCATGGCCGTGTCCGAGCTCGAGGTCGACGGCGTCGTCTTCCCGCCGCTCGCCCGCCCGCCGG

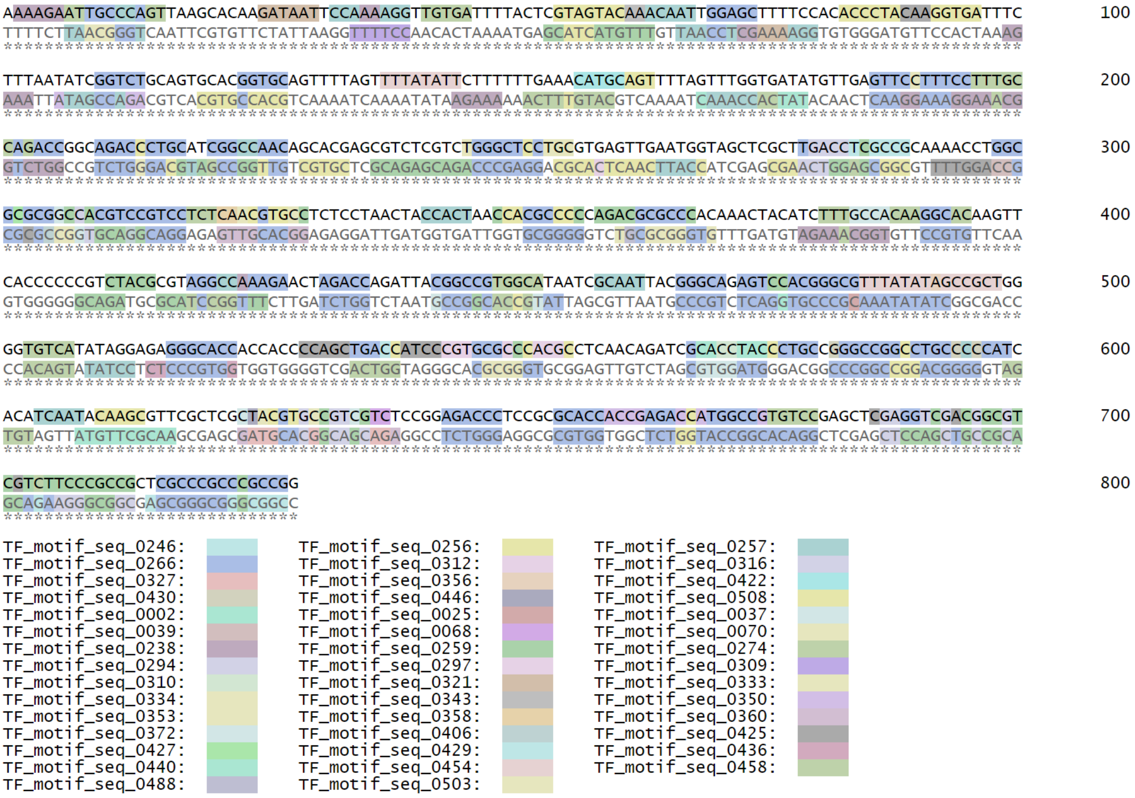

Factor or Site Name	Loc.(Str.)	Signal Sequence	SITE #
DOFCOREZM	2 (+)	AAAG	S000265
EECCRCAH1	5 (+)	GANTTNC	S000494
CAATBOX1	7 (-)	CAAT	S000028
MYB2AT	13 (-)	TAACTG	S000177

MYB2CONSENSUSAT	13	(-)	YAACKG	S000409
MYBCORE	13	(+)	CNGTTR	S000176
GATABOX	26	(+)	GATA	S000039
GT1CONSENSUS	26	(+)	GRWAAW	S000198
IBOXCORE	26	(+)	GATAA	S000199
PYRIMIDINEBOXOSRAMY1A	35	(-)	CCTTTT	S000259
DOFCOREZM	36	(+)	AAAG	S000265
GTGANTG10	43	(+)	GTGA	S000378
ARR1AT	44	(+)	NGATT	S000454
CACTFTPPCA1	50	(+)	YACT	S000449
CACTFTPPCA1	57	(-)	YACT	S000449
CURECORECR	58	(-)	GTAC	S000493
CURECORECR	58	(+)	GTAC	S000493
EBOXBNNAPA	65	(-)	CANNTG	S000144
MYCCONSENSUSAT	65	(-)	CANNTG	S000407
CAATBOX1	65	(+)	CAAT	S000028
EBOXBNNAPA	65	(+)	CANNTG	S000144
MYCCONSENSUSAT	65	(+)	CANNTG	S000407
CAATBOX1	67	(-)	CAAT	S000028
CCAATBOX1	67	(-)	CCAAT	S000030
DOFCOREZM	74	(-)	AAAG	S000265
GT1CONSENSUS	75	(-)	GRWAAW	S000198
GTGANTG10	93	(+)	GTGA	S000378
ARR1AT	94	(+)	NGATT	S000454
POLLEN1LELAT52	97	(-)	AGAAA	S000245
DOFCOREZM	100	(-)	AAAG	S000265
TAAAGSTKST1	100	(-)	TAAAG	S000387
ROOTMOTIFTAPOX1	104	(-)	ATATT	S000098
GATABOX	106	(-)	GATA	S000039
CACTFTPPCA1	117	(-)	YACT	S000449
ROOTMOTIFTAPOX1	141	(+)	ATATT	S000098
-10PEHVPSBD	142	(+)	TATTCT	S000392
DOFCOREZM	146	(-)	AAAG	S000265
RYREPEATBNNAPA	157	(+)	CATGCA	S000264
MYBPLANT	169	(-)	MACCWAMC	S000167
GTGANTG10	174	(+)	GTGA	S000378
GATABOX	176	(+)	GATA	S000039
RAV1AAT	180	(-)	CAACA	S000314
EECCRCAH1	184	(+)	GANTTNC	S000494
DOFCOREZM	190	(-)	AAAG	S000265
DOFCOREZM	195	(-)	AAAG	S000265
INTRONLOWER	214	(-)	TGCAGG	S000086
MYBCORE	225	(-)	CNGTTR	S000176
RAV1AAT	225	(+)	CAACA	S000314
RHERPATEXPA7	230	(+)	KCACGW	S000512
SURECOREATSULTR11	238	(-)	GAGAC	S000499
SITEIIATCYTC	246	(+)	TGGGCY	S000474
GTGANTG10	257	(+)	GTGA	S000378
CAREOSREP1	259	(-)	CAACTC	S000421
S1FBOXSORPS1L21	266	(+)	ATGGTA	S000223
ELRECOREPCRP1	278	(+)	TTGACC	S000142
WBOXATNPR1	278	(+)	TTGAC	S000390
QELEMENTZMZM13	279	(-)	AGGTCA	S000254
WRKY71OS	279	(+)	TGAC	S000447
WBOXNTERF3	279	(+)	TGACY	S000457
CGCGBOXAT	299	(-)	VCGCGB	S000501
CGCGBOXAT	299	(+)	VCGCGB	S000501
CGCGBOXAT	301	(-)	VCGCGB	S000501

CGCGBOXAT	301	(+)	VCGCGB	S000501
ABREOSRAB21	305	(-)	ACGTSSSC	S000012
BOXIIPCCHS	306	(-)	ACGTGGC	S000229
ACGTABREMOTIFA2OSEM	306	(-)	ACGTGKC	S000394
SORLIP1AT	306	(+)	GCCAC	S000482
ABRELATERD1	308	(-)	ACGTG	S000414
ACGTATERD1	309	(-)	ACGT	S000415
ACGTATERD1	309	(+)	ACGT	S000415
PALBOXAPC	313	(+)	CCGTCC	S000137
BP5OSWX	322	(+)	CAACGTG	S000436
T/GBOXATPIN2	323	(+)	AACGTG	S000458
ABRERATCAL	323	(+)	MACGYGB	S000507
ACGTATERD1	324	(-)	ACGT	S000415
RHERPATEXPA7	324	(-)	KCACGW	S000512
ABRELATERD1	324	(+)	ACGTG	S000414
ACGTATERD1	324	(+)	ACGT	S000415
CACTFTPPCA1	343	(+)	YACT	S000449
SV40COREENHAN	345	(-)	GTGGWWHG	S000123
MYBATRD22	345	(+)	CTAACCA	S000175
MYB1AT	346	(+)	WAACCA	S000408
CGCGBOXAT	361	(-)	VCGCGB	S000501
CGCGBOXAT	361	(+)	VCGCGB	S000501
NODCON1GM	378	(-)	AAAGAT	S000461
OSE1ROOTNODULE	378	(-)	AAAGAT	S000467
DOFCOREZM	380	(-)	AAAG	S000265
SORLIP1AT	384	(+)	GCCAC	S000482
GTGANTG10	400	(-)	GTGA	S000378
CGCGBOXAT	413	(-)	VCGCGB	S000501
CGCGBOXAT	413	(+)	VCGCGB	S000501
DOFCOREZM	424	(+)	AAAG	S000265
PRECONSCRHSP70A	437	(-)	SCGAYNRNNNNNNNNNNNNNNNHD	S000506
ARR1AT	437	(+)	NGATT	S000454
SORLIP1AT	448	(-)	GCCAC	S000482
ARR1AT	455	(-)	NGATT	S000454
CAATBOX1	460	(+)	CAAT	S000028
TATAPVTRNALEU	485	(+)	TTTATATA	S000340
TATABOX4	486	(-)	TATATAA	S000111
BIHD1OS	503	(+)	TGTCA	S000498
WRKY71OS	504	(-)	TGAC	S000447
EBOXBNNAPA	531	(-)	CANNTG	S000144
MYCCONSENSUSAT	531	(-)	CANNTG	S000407
EBOXBNNAPA	531	(+)	CANNTG	S000144
MYCCONSENSUSAT	531	(+)	CANNTG	S000407
WBOXNTCHN48	534	(+)	CTGACY	S000508
WRKY71OS	535	(+)	TGAC	S000447
WBOXNTERF3	535	(+)	TGACY	S000457
BOXLCOREDCPAL	537	(+)	ACCWWCC	S000492
**MYBCORE**	**559**	**(-)**	**CNGTTR**	**S000176**
RAV1AAT	559	(+)	CAACA	S000314
MYBPLANT	569	(+)	MACCWAMC	S000167
BOXLCOREDCPAL	570	(+)	ACCWWCC	S000492
MYBPZM	571	(+)	CCWACC	S000179
**SORLIP2AT**	**582**	**(+)**	**GGGCC**	**S000483**
GTGANTG10	599	(-)	GTGA	S000378
CAATBOX1	605	(+)	CAAT	S000028
ACGTATERD1	626	(-)	ACGT	S000415
RHERPATEXPA7	626	(-)	KCACGW	S000512
**ABRELATERD1**	**626**	**(+)**	**ACGTG**	**S000414**
**ACGTATERD1**	**626**	**(+)**	**ACGT**	**S000415**

HEXAMERATH4	631 (+)	CCGTCG	S000146
**CGACGOSAMY3**	**632 (-)**	**CGACG**	**S000205**
SURECOREATSULTR11	636 (-)	GAGAC	S000499
**SURECOREATSULTR11**	**643 (+)**	**GAGAC**	**S000499**
**CGCGBOXAT**	**651 (-)**	**VCGCGB**	**S000501**
**CGCGBOXAT**	**651 (+)**	**VCGCGB**	**S000501**
DRE1COREZMRAB17	660 (+)	ACCGAGA	S000401
SURECOREATSULTR11	663 (+)	GAGAC	S000499
**CRTDREHVCBF2**	**690 (-)**	**GTCGAC**	**S000411**
**CBFHV**	**690 (-)**	**RYCGAC**	**S000497**
**CRTDREHVCBF2**	**690 (+)**	**GTCGAC**	**S000411**
**CBFHV**	**690 (+)**	**RYCGAC**	**S000497**
**HEXAMERATH4**	**692 (-)**	**CCGTCG**	**S000146**
**CGACGOSAMY3**	**692 (+)**	**CGACG**	**S000205**
CGACGOSAMY3	698 (-)	CGACG	S000205

//

>TaChi-B1

```
CGGACCCAAACGGACAAAAAGCGAACAAAATCACCGTCCGTTTGGGTCGGCACCGTTGGAGTTGCTCTAATCCTTATTAAT
TCATGCCTGAGGTAGGCGGAACATTACTCTTGCCTACCCCGTAGGGTGGTTTCTTTTGAGATCGGTGTGCTTTGGTGACAT
GTTGAGCCGTTGTGTCTGGTGTGGGCTCGTGCGTGAGTTGAATGGTAGCTTGACGTCGCCGCAAAACCTGGCGCGCGGCCA
CGTCCGTCCTCTCTTTGCCCCTCAACGTGCCTCTCCCAACTACCACTAACCACGCCCCCATATGCGCACACAAACTACATC
TTTGCCACAAGGCACAAGGTCCACCCTCCGTTACCGTCTAGTCTAGGCCGCGTAGGCCAGAGAACCAGACCAGATTACAGC
CGTGGCACAATCGCAATTACGGGCAGAGTGTACGGGCGTTTATATAGCTGTTGCGGTGTCATATAGGGGGGCACGCACCA
CCACCCCAGCTACCCATCCCGTGCGCCCACGCCTCGCCAGATCGCACCTACCCTGCCGCCCTGCCCCCGTCCCATCAATAC
AAGTGTTTGCTCGCTACGTGCCGTCCGTCGTCTCCGGAGACCCTCCATCCATCCATCCGGCGATATACC
```

```
CGGACCCAAACGGACAAAAAGCGAACAAAATCACCGTCCGTTTGGGTCGGCACCGTTGGAGTTGCTCTAATCCTTATTAATTCATGCCTGAGGTAGGCGG   100
GCCTGGGTTTGCCTGTTTTTCGCTTGTTTTAGTGGCAGGCAAACCCAGCCGTGGCAACCTCAACGAGATTAGGAATAATTAAGTACGGACTCCATCCGCC
****************************************************************************************************

AACATTACTCTTGCCTACCCCGTAGGGTGGTTTCTTTTGAGATCGGTGTGCTTTGGTGACATGTTGAGCCGTTGTGTCTGGTGTGGGCTCGTGCGTGAGT   200
TTGTAATGAGAACGGATGGGGCATCCCACCAAAGAAAACTCTAGCCACACGAAACCACTGTACAACTCGGCAACACAGACCACACCCGAGCACGCACTCA
****************************************************************************************************

TGAATGGTAGCTTGACGTCGCCGCAAAACCTGGCGCGCGGCCACGTCCGTCCTCTCTTTGCCCCTCAACGTGCCTCTCCCAACTACCACTAACCACGCCC   300
ACTTACCATCGAACTGCAGCGGCGTTTTGGACCGCGCGCCGGTGCAGGCAGGAGAGAAACGGGGAGTTGCACGGAGAGGGTTGATGGTGATTGGTGCGGG
****************************************************************************************************

CCATATGCGCACACAAACTACATCTTTGCCACAAGGCACAAGGTCCACCCTCCGTTACCGTCTAGTCTAGGCCGCGTAGGCCAGAGAACCAGACCAGATT   400
GGTATACGCGTGTGTTTGATGTAGAAACGGTGTTCCGTGTTCCAGGTGGGAGGCAATGGCAGATCAGATCCGGCGCATCCGGTCTCTTGGTCTGGTCTAA
****************************************************************************************************

ACAGCCGTGGCACAATCGCAATTACGGGCAGAGTGTACGGGCGTTTATATAGCTGTTGCGGTGTCATATAGGGGGGCACGCACCACCACCCCAGCTACC   500
TGTCGGCACCGTGTTAGCGTTAATGCCCGTCTCACATGCCCGCAAATATATCGACAACGCCACAGTATATCCCCCCGTGCGTGGTGGTGGGGTCGATGG
****************************************************************************************************

CATCCCGTGCGCCCACGCCTCGCCAGATCGCACCTACCCTGCCGCCCTGCCCCCGTCCCATCAATACAAGTGTTTGCTCGCTACGTGCCGTCCGTCGTCT   600
GTAGGGCACGCGGGTGCGGAGCGGTCTAGCGTGGATGGGACGGCGGGACGGGGGCAGGGTAGTTATGTTCACAAACGAGCGATGCACGGCAGGCAGCAGA
****************************************************************************************************

CCGGAGACCCTCCATCCATCCATCCGGCGATATACC                                                                  700
GGCCTCTGGGAGGTAGGTAGGTAGGCCGCTATATGG
************************************
```

TF_motif_seq_0246:	TF_motif_seq_0256:	TF_motif_seq_0257:
TF_motif_seq_0266:	TF_motif_seq_0292:	TF_motif_seq_0312:
TF_motif_seq_0316:	TF_motif_seq_0327:	TF_motif_seq_0347:
TF_motif_seq_0422:	TF_motif_seq_0431:	TF_motif_seq_0446:
TF_motif_seq_0508:	TF_motif_seq_0002:	TF_motif_seq_0025:
TF_motif_seq_0031:	TF_motif_seq_0037:	TF_motif_seq_0039:
TF_motif_seq_0068:	TF_motif_seq_0121:	TF_motif_seq_0132:
TF_motif_seq_0238:	TF_motif_seq_0259:	TF_motif_seq_0274:
TF_motif_seq_0283:	TF_motif_seq_0297:	TF_motif_seq_0310:
TF_motif_seq_0324:	TF_motif_seq_0334:	TF_motif_seq_0344:
TF_motif_seq_0353:	TF_motif_seq_0358:	TF_motif_seq_0360:
TF_motif_seq_0372:	TF_motif_seq_0380:	TF_motif_seq_0406:
TF_motif_seq_0425:	TF_motif_seq_0427:	TF_motif_seq_0429:
TF_motif_seq_0440:	TF_motif_seq_0454:	TF_motif_seq_0458:
TF_motif_seq_0488:	TF_motif_seq_0503:	

Factor or Site Name	Loc.(Str.)	Signal Sequence	SITE #
MYBCOREATCYCB1	9 (+)	AACGG	S000502
DOFCOREZM	18 (+)	AAAG	S000265
ARR1AT	29 (-)	NGATT	S000454

GTGANTG10	31	(-)	GTGA	S000378
**PALBOXAPC**	**34**	**(+)**	**CCGTCC**	**S000137**
MYBCOREATCYCB1	38	(-)	AACGG	S000502
**LTRECOREATCOR15**	**46**	**(-)**	**CCGAC**	**S000153**
**DRECRTCOREAT**	**46**	**(-)**	**RCCGAC**	**S000418**
**CBFHV**	**46**	**(-)**	**RYCGAC**	**S000497**
**MYB2CONSENSUSAT**	**53**	**(-)**	**YAACKG**	**S000409**
**MYBCOREATCYCB1**	**53**	**(-)**	**AACGG**	**S000502**
**MYBCORE**	**53**	**(+)**	**CNGTTR**	**S000176**
CAREOSREP1	59	(-)	CAACTC	S000421
EECCRCAH1	59	(+)	GANTTNC	S000494
ARR1AT	69	(-)	NGATT	S000454
TATABOX3	75	(+)	TATTAAT	S000110
MYBPZM	92	(-)	CCWACC	S000179
CACTFTPPCA1	106	(+)	YACT	S000449
NODCON2GM	108	(+)	CTCTT	S000462
OSE2ROOTNODULE	108	(+)	CTCTT	S000468
MYBPZM	114	(+)	CCWACC	S000179
MYB1AT	128	(-)	WAACCA	S000408
POLLEN1LELAT52	131	(-)	AGAAA	S000245
DOFCOREZM	134	(-)	AAAG	S000265
DOFCOREZM	151	(-)	AAAG	S000265
GTGANTG10	156	(+)	GTGA	S000378
BIHD1OS	157	(-)	TGTCA	S000498
WRKY71OS	157	(+)	TGAC	S000447
RAV1AAT	162	(-)	CAACA	S000314
MYB2CONSENSUSAT	169	(-)	YAACKG	S000409
MYBCOREATCYCB1	169	(-)	AACGG	S000502
MYBCORE	169	(+)	CNGTTR	S000176
DPBFCOREDCDC3	178	(-)	ACACNNG	S000292
SITEIIATCYTC	184	(+)	TGGGCY	S000474
RHERPATEXPA7	189	(-)	KCACGW	S000512
GTGANTG10	195	(+)	GTGA	S000378
CAREOSREP1	197	(-)	CAACTC	S000421
S1FBOXSORPS1L21	204	(+)	ATGGTA	S000223
WBOXATNPR1	212	(+)	TTGAC	S000390
HEXMOTIFTAH3H4	213	(-)	ACGTCA	S000053
ASF1MOTIFCAMV	213	(+)	TGACG	S000024
TGACGTVMAMY	213	(+)	TGACGT	S000377
WRKY71OS	213	(+)	TGAC	S000447
ACGTCBOX	214	(-)	GACGTC	S000131
ACGTCBOX	214	(+)	GACGTC	S000131
ACGTATERD1	215	(-)	ACGT	S000415
ABREOSRAB21	215	(+)	ACGTSSSC	S000012
ACGTATERD1	215	(+)	ACGT	S000415
**CGACGOSAMY3**	**216**	**(-)**	**CGACG**	**S000205**
CGCGBOXAT	233	(-)	VCGCGB	S000501
CGCGBOXAT	233	(+)	VCGCGB	S000501
CGCGBOXAT	235	(-)	VCGCGB	S000501
CGCGBOXAT	235	(+)	VCGCGB	S000501
ABREOSRAB21	239	(-)	ACGTSSSC	S000012
BOXIIPCCHS	240	(-)	ACGTGGC	S000229
ACGTABREMOTIFA2OSEM	240	(-)	ACGTGKC	S000394
SORLIP1AT	240	(+)	GCCAC	S000482
ABRELATERD1	242	(-)	ACGTG	S000414
ACGTATERD1	243	(-)	ACGT	S000415
ACGTATERD1	243	(+)	ACGT	S000415
PALBOXAPC	247	(+)	CCGTCC	S000137
NODCON2GM	254	(+)	CTCTT	S000462

OSE2ROOTNODULE	254	(+)	CTCTT	S000468
DOFCOREZM	256	(-)	AAAG	S000265
E2FCONSENSUS	257	(+)	WTTSSCSS	S000476
BP5OSWX	266	(+)	CAACGTG	S000436
T/GBOXATPIN2	267	(+)	AACGTG	S000458
ABRERATCAL	267	(+)	MACGYGB	S000507
ACGTATERD1	268	(-)	ACGT	S000415
RHERPATEXPA7	268	(-)	KCACGW	S000512
ABRELATERD1	268	(+)	ACGTG	S000414
ACGTATERD1	268	(+)	ACGT	S000415
UPRMOTIFIIAT	279	(+)	CCNNNNNNNNNNNNCCACG	S000426
CACTFTPPCA1	287	(+)	YACT	S000449
SV40COREENHAN	289	(-)	GTGGWWHG	S000123
MYBATRD22	289	(+)	CTAACCA	S000175
MYB1AT	290	(+)	WAACCA	S000408
EBOXBNNAPA	302	(-)	CANNTG	S000144
CATATGGMSAUR	302	(-)	CATATG	S000370
MYCCONSENSUSAT	302	(-)	CANNTG	S000407
EBOXBNNAPA	302	(+)	CANNTG	S000144
CATATGGMSAUR	302	(+)	CATATG	S000370
MYCCONSENSUSAT	302	(+)	CANNTG	S000407
NODCON1GM	322	(-)	AAAGAT	S000461
OSE1ROOTNODULE	322	(-)	AAAGAT	S000467
DOFCOREZM	324	(-)	AAAG	S000265
SORLIP1AT	328	(+)	GCCAC	S000482
MYB2CONSENSUSAT	352	(-)	YAACKG	S000409
MYBCOREATCYCB1	352	(-)	AACGG	S000502
MYBCORE	352	(+)	CNGTTR	S000176
CGCGBOXAT	372	(-)	VCGCGB	S000501
CGCGBOXAT	372	(+)	VCGCGB	S000501
PRECONSCRHSP70A	396	(-)	SCGAYNRNNNNNNNNNNNNNNNHD	S000506
ARR1AT	396	(+)	NGATT	S000454
SORLIP1AT	407	(-)	GCCAC	S000482
CAATBOX1	413	(+)	CAAT	S000028
ARR1AT	414	(-)	NGATT	S000454
CAATBOX1	419	(+)	CAAT	S000028
CACTFTPPCA1	432	(-)	YACT	S000449
CURECORECR	435	(-)	GTAC	S000493
CURECORECR	435	(+)	GTAC	S000493
TATAPVTRNALEU	444	(+)	TTTATATA	S000340
TATABOX4	445	(-)	TATATAA	S000111
MYBCORE	453	(+)	CNGTTR	S000176
RAV1AAT	454	(-)	CAACA	S000314
BIHD1OS	462	(+)	TGTCA	S000498
WRKY71OS	463	(-)	TGAC	S000447
UPRMOTIFIIAT	499	(+)	CCNNNNNNNNNNNNCCACG	S000426
MYBPLANT	531	(+)	MACCWAMC	S000167
BOXLCOREDCPAL	532	(+)	ACCWWCC	S000492
MYBPZM	533	(+)	CCWACC	S000179
GCCCORE	541	(+)	GCCGCC	S000430
PALBOXAPC	553	(+)	CCGTCC	S000137
CAATBOX1	562	(+)	CAAT	S000028
EBOXBNNAPA	567	(-)	CANNTG	S000144
DPBFCOREDCDC3	567	(-)	ACACNNG	S000292
MYCCONSENSUSAT	567	(-)	CANNTG	S000407
EBOXBNNAPA	567	(+)	CANNTG	S000144
MYCCONSENSUSAT	567	(+)	CANNTG	S000407
CACTFTPPCA1	569	(-)	YACT	S000449

2SSEEDPROTBANAPA	570 (-)	CAAACAC	S000143
CANBNNAPA	570 (-)	CNAACAC	S000148
ACGTATERD1	583 (-)	ACGT	S000415
RHERPATEXPA7	583 (-)	KCACGW	S000512
ABRELATERD1	583 (+)	ACGTG	S000414
ACGTATERD1	583 (+)	ACGT	S000415
PALBOXAPC	588 (+)	CCGTCC	S000137
HEXAMERATH4	592 (+)	CCGTCG	S000146
**CGACGOSAMY3**	**593 (-)**	**CGACG**	**S000205**
SURECOREATSULTR11	597 (-)	GAGAC	S000499
SURECOREATSULTR11	604 (+)	GAGAC	S000499
GATABOX	629 (+)	GATA	S000039

//

>TaChi-D1
GGAGCTTTTCCACACCCTATAAGGTGGTTTCTTTAATTCTGTTGAGATCGGTCTGCAGTGCACGGTGCAGTTTTTGTTTTT
GTTTTTTGAAACATTCAGTTTTAGTTTGGTGATATGTTGAGTTCCTTTGCTTTACCAGACCGGCAGACCCTGCATCGGCCA
GCAACACGAGTGTCTGGTCTGGACTCGTGCGTGAGTTTTTTTTTTTTTTGAGGGACTCGTGCGTGAGTTGAATGGTAGCT
TGACCTCGCCGCAAAACCTGGCGCGCGGACACGTCCTCTCTCTGCCCCTCAACGTGCCTCTCCTAACTACCACTAACCACG
CCCCCAGATGCGCCCACAAACTACATCTCTGCCACAAGGACAAGGTCCACCCCCCGTCACCGTCTAGTCTAGGCCGCGTAG
GCCAGAGAACCAGACCAGATTACGGCCGTGGCATAATCGCAATTACGGGCAGAGTCGACGGGCGTTTATATAGCTGTTGCG
GTGTCATATAGGGGGGGCACGCACCACCACCCCAGCTACCCATCCCGTGCGCCTACGCCTCGCCAGATCGCACCTACCCTG
CCGGCCTGCCCCCATCACATCAATACAAGTGTTCGCTCGCTACGTGCCGTCGTCTCCGGAGACCCTCCGTGCACCACC

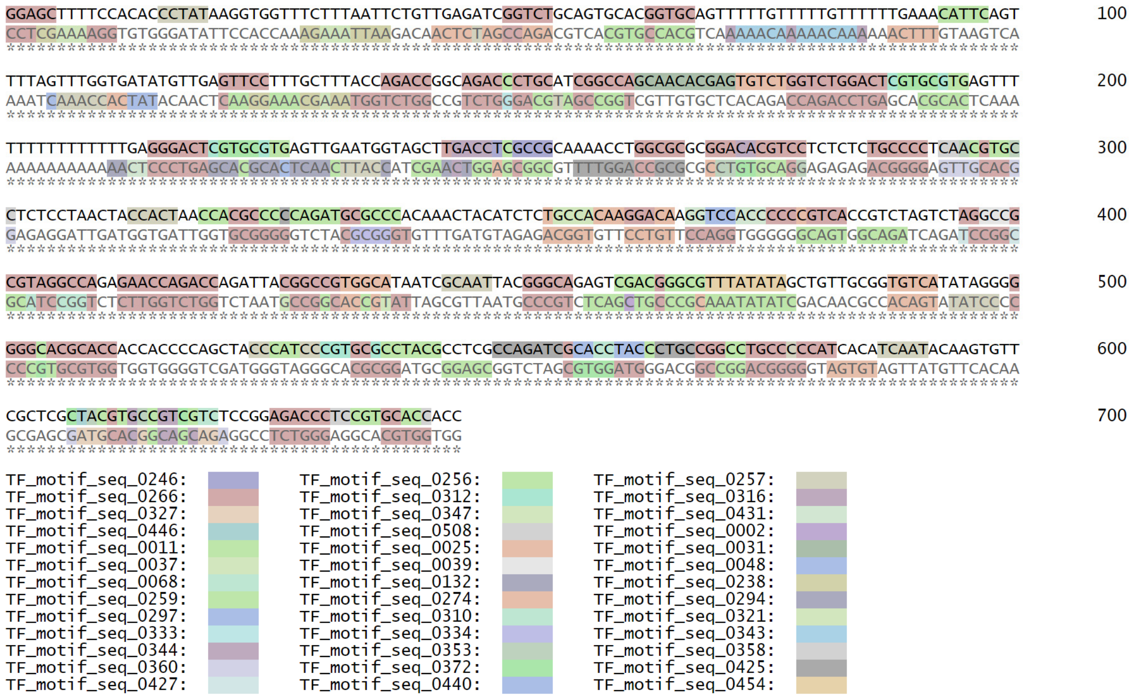

Factor or Site Name	Loc.(Str.)	Signal Sequence	SITE #
DOFCOREZM	5 (-)	AAAG	S000265
GT1CONSENSUS	6 (-)	GRWAAW	S000198
MYB1AT	25 (-)	WAACCA	S000408
POLLEN1LELAT52	28 (-)	AGAAA	S000245
DOFCOREZM	31 (-)	AAAG	S000265
TAAAGSTKST1	31 (-)	TAAAG	S000387
POLASIG2	32 (-)	AATTAAA	S000081

MYBCORE	39	(+)	CNGTTR	S000176
RAV1AAT	40	(-)	CAACA	S000314
CACTFTPPCA1	57	(-)	YACT	S000449
SEF4MOTIFGM7S	70	(+)	RTTTTTR	S000103
ANAERO1CONSENSUS	73	(-)	AAACAAA	S000477
SEF4MOTIFGM7S	76	(+)	RTTTTTR	S000103
ANAERO1CONSENSUS	79	(-)	AAACAAA	S000477
INRNTPSADB	95	(+)	YTCANTYY	S000395
MYBPLANT	105	(-)	MACCWAMC	S000167
GTGANTG10	110	(+)	GTGA	S000378
GATABOX	112	(+)	GATA	S000039
RAV1AAT	116	(-)	CAACA	S000314
EECCRCAH1	120	(+)	GANTTNC	S000494
DOFCOREZM	126	(-)	AAAG	S000265
DOFCOREZM	131	(-)	AAAG	S000265
TAAAGSTKST1	131	(-)	TAAAG	S000387
GT1CONSENSUS	132	(-)	GRWAAW	S000198
INTRONLOWER	150	(-)	TGCAGG	S000086
RAV1AAT	164	(+)	CAACA	S000314
DPBFCOREDCDC3	166	(+)	ACACNNG	S000292
DPBFCOREDCDC3	169	(-)	ACACNNG	S000292
CACTFTPPCA1	171	(-)	YACT	S000449
RHERPATEXPA7	187	(-)	KCACGW	S000512
GTGANTG10	193	(+)	GTGA	S000378
MARTBOX	198	(+)	TTWTWTTWTT	S000067
MARTBOX	199	(+)	TTWTWTTWTT	S000067
MARTBOX	200	(+)	TTWTWTTWTT	S000067
MARTBOX	201	(+)	TTWTWTTWTT	S000067
MARTBOX	202	(+)	TTWTWTTWTT	S000067
MARTBOX	203	(+)	TTWTWTTWTT	S000067
RHERPATEXPA7	220	(-)	KCACGW	S000512
GTGANTG10	226	(+)	GTGA	S000378
CAREOSREP1	228	(-)	CAACTC	S000421
S1FBOXSORPS1L21	235	(+)	ATGGTA	S000223
ELRECOREPCRP1	243	(+)	TTGACC	S000142
WBOXATNPR1	243	(+)	TTGAC	S000390
QELEMENTZMZM13	244	(-)	AGGTCA	S000254
WRKY71OS	244	(+)	TGAC	S000447
WBOXNTERF3	244	(+)	TGACY	S000457
CGCGBOXAT	264	(-)	VCGCGB	S000501
CGCGBOXAT	264	(+)	VCGCGB	S000501
CGCGBOXAT	266	(-)	VCGCGB	S000501
CGCGBOXAT	266	(+)	VCGCGB	S000501
ACGTABREMOTIFA2OSEM	271	(-)	ACGTGKC	S000394
GADOWNAT	271	(-)	ACGTGTC	S000438
ABRELATERD1	273	(-)	ACGTG	S000414
ACGTATERD1	274	(-)	ACGT	S000415
ACGTATERD1	274	(+)	ACGT	S000415
BP5OSWX	293	(+)	CAACGTG	S000436
T/GBOXATPIN2	294	(+)	AACGTG	S000458
ABRERATCAL	294	(+)	MACGYGB	S000507
ACGTATERD1	295	(-)	ACGT	S000415
RHERPATEXPA7	295	(-)	KCACGW	S000512
ABRELATERD1	295	(+)	ACGTG	S000414
ACGTATERD1	295	(+)	ACGT	S000415
CACTFTPPCA1	314	(+)	YACT	S000449
SV40COREENHAN	316	(-)	GTGGWWHG	S000123
MYBATRD22	316	(+)	CTAACCA	S000175

MYB1AT	317	(+)	WAACCA	S000408
EBOXBNNAPA	329	(-)	CANNTG	S000144
MYCCONSENSUSAT	329	(-)	CANNTG	S000407
EBOXBNNAPA	329	(+)	CANNTG	S000144
MYCCONSENSUSAT	329	(+)	CANNTG	S000407
SORLIP1AT	355	(+)	GCCAC	S000482
ASF1MOTIFCAMV	379	(-)	TGACG	S000024
WRKY71OS	380	(-)	TGAC	S000447
GTGANTG10	381	(-)	GTGA	S000378
CGCGBOXAT	398	(-)	VCGCGB	S000501
CGCGBOXAT	398	(+)	VCGCGB	S000501
PRECONSCRHSP70A	422	(-)	SCGAYNRNNNNNNNNNNNNNNNHD	S000506
ARR1AT	422	(+)	NGATT	S000454
SORLIP1AT	433	(-)	GCCAC	S000482
ARR1AT	440	(-)	NGATT	S000454
CAATBOX1	445	(+)	CAAT	S000028
CRTDREHVCBF2	459	(-)	GTCGAC	S000411
CBFHV	459	(-)	RYCGAC	S000497
CRTDREHVCBF2	459	(+)	GTCGAC	S000411
CBFHV	459	(+)	RYCGAC	S000497
HEXAMERATH4	461	(-)	CCGTCG	S000146
CGACGOSAMY3	461	(+)	CGACG	S000205
TATAPVTRNALEU	470	(+)	TTTATATA	S000340
TATABOX4	471	(-)	TATATAA	S000111
MYBCORE	479	(+)	CNGTTR	S000176
RAV1AAT	480	(-)	CAACA	S000314
BIHD1OS	488	(+)	TGTCA	S000498
WRKY71OS	489	(-)	TGAC	S000447
MYBPLANT	557	(+)	MACCWAMC	S000167
BOXLCOREDCPAL	558	(+)	ACCWWCC	S000492
MYBPZM	559	(+)	CCWACC	S000179
GTGANTG10	582	(-)	GTGA	S000378
CAATBOX1	588	(+)	CAAT	S000028
EBOXBNNAPA	593	(-)	CANNTG	S000144
DPBFCOREDCDC3	593	(-)	ACACNNG	S000292
MYCCONSENSUSAT	593	(-)	CANNTG	S000407
EBOXBNNAPA	593	(+)	CANNTG	S000144
MYCCONSENSUSAT	593	(+)	CANNTG	S000407
CACTFTPPCA1	595	(-)	YACT	S000449
CANBNNAPA	596	(-)	CNAACAC	S000148
ACGTATERD1	609	(-)	ACGT	S000415
RHERPATEXPA7	609	(-)	KCACGW	S000512
ABRELATERD1	609	(+)	ACGTG	S000414
ACGTATERD1	609	(+)	ACGT	S000415
HEXAMERATH4	614	(+)	CCGTCG	S000146
CGACGOSAMY3	615	(-)	CGACG	S000205
SURECOREATSULTR11	619	(-)	GAGAC	S000499
SURECOREATSULTR11	626	(+)	GAGAC	S000499

//

>TaF3H-B1
TCTACAAGCCATGGAAATATGACGTGTCATGATGTATAGGTTTTAGATATTGGGCGAACCAATCTGTGGTTGGATGGTTAA
AGGGACTCTGATATCCCCAGTCCACTAAGGTTCAAATCTGGTGCTTGAGTTTATTCCTGGATTATTTCAGGATTTTCGACG
ATGTGCATTCAGTAGGAGAAGACGTTCCCGTCGACAACGAGTTGCCCACGATGACTTCGTAAATTTCAAGATAATATGCCG
ATTCGGTCTTTCGGAGGTGCTCATAGGGGTAGGGTGTGCGTGTGTGCGTTTATAGGGATGAGTGTATGCACGTATATATGA
GCGCTTGTATCTGGACTGTGTTAAAAAAAAGTTCTAGATCTTGGTTGGTGTATAGCGGGTGTCCAGTGGTGTGTGTGCTTG
TGATGTGTGTGTTGTGCGTATATGGATCGATCGATACGGCTGGATGGATGCATGGGTGCGGCCCGGTTAGTTGCGCACCTA
ACACGAGTGCCCGAGGGCGACTTCCTTGGTTAGTTGCGTGCGCACGCGCGACTTGCAGGTCGCGTCTGCCCCTCATCTTCC

AACGTAGTATACACAATGCCCTCTTCCCGGCCGTATATATCCGCGTGCGTACAACGTCCAGAAAACCACAACCCAAACGGC
CGAGCCTTTCATCAAGGAACAAACCATCATACGAGACAGACGCCGGCCGCGACACAAGCGGACGATCCCCGGAGTGCGGAT
CGATGGCGCCGGTGAGCAACGAGACGTTCCTCCCGA

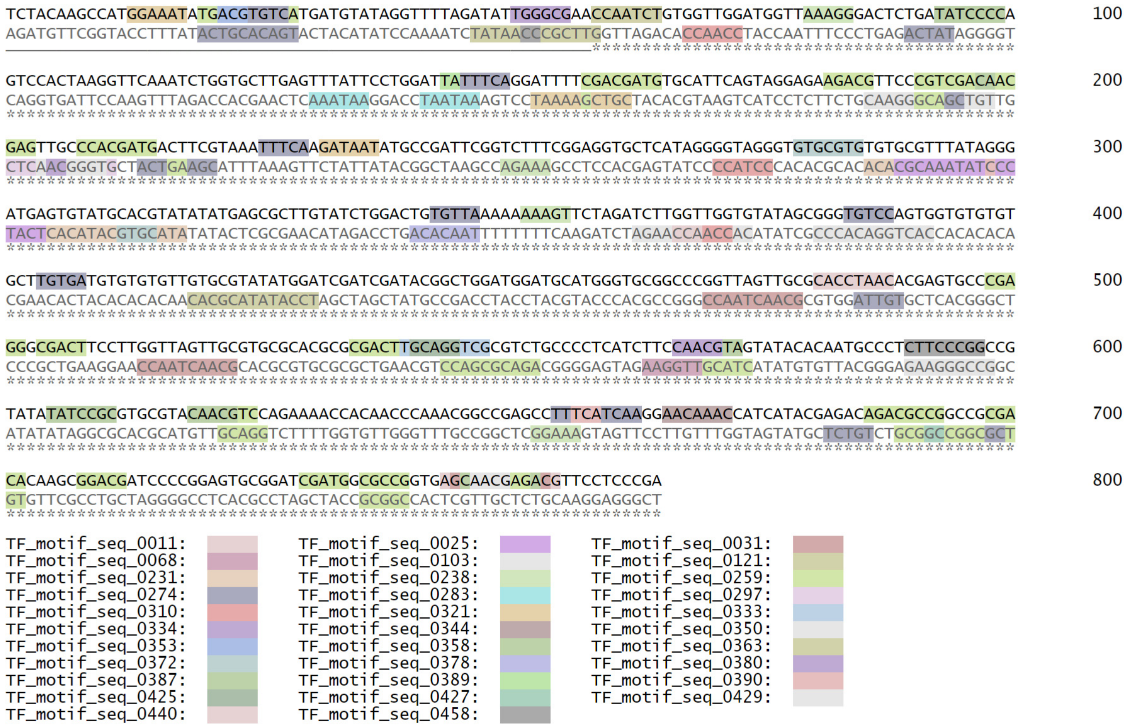

Factor or Site Name	Loc.(Str.)	Signal Sequence	SITE #
GT1CONSENSUS	13 (+)	GRWAAW	S000198
ROOTMOTIFTAPOX1	16 (-)	ATATT	S000098
HEXMOTIFTAH3H4	20 (-)	ACGTCA	S000053
ASF1MOTIFCAMV	20 (+)	TGACG	S000024
TGACGTVMAMY	20 (+)	TGACGT	S000377
WRKY71OS	20 (+)	TGAC	S000447
ACGTATERD1	22 (-)	ACGT	S000415
ACGTABREMOTIFA2OSEM	22 (+)	ACGTGKC	S000394
ABRELATERD1	22 (+)	ACGTG	S000414
ACGTATERD1	22 (+)	ACGT	S000415
GADOWNAT	22 (+)	ACGTGTC	S000438
BIHD1OS	25 (+)	TGTCA	S000498
WRKY71OS	26 (-)	TGAC	S000447
GATABOX	46 (+)	GATA	S000039
ROOTMOTIFTAPOX1	47 (+)	ATATT	S000098
CAATBOX1	49 (-)	CAAT	S000028
CCAATBOX1	49 (-)	CCAAT	S000030
REALPHALGLHCB21	57 (+)	AACCAA	S000362
CCAATBOX1	59 (+)	CCAAT	S000030
CAATBOX1	60 (+)	CAAT	S000028
ARR1AT	61 (-)	NGATT	S000454
SV40COREENHAN	66 (+)	GTGGWWHG	S000123
BOXLCOREDCPAL	73 (-)	ACCWWCC	S000492
MYB1AT	76 (-)	WAACCA	S000408
GT1CORE	77 (+)	GGTTAA	S000125
TAAAGSTKST1	80 (+)	TAAAG	S000387

DOFCOREZM	81	(+)	AAAG	S000265
GATABOX	92	(+)	GATA	S000039
**GATABOX**	**94**	**(-)**	**GATA**	**S000039**
**MYBST1**	**94**	**(-)**	**GGATA**	**S000180**
CACTFTPPCA1	105	(+)	YACT	S000449
ARR1AT	117	(-)	NGATT	S000454
POLASIG1	132	(-)	AATAAA	S000080
POLASIG3	144	(-)	AATAAT	S000088
TATABOX5	145	(+)	TTATTT	S000203
ARR1AT	153	(+)	NGATT	S000454
EECCRCAH1	154	(+)	GANTTNC	S000494
GT1CONSENSUS	155	(-)	GRWAAW	S000198
CGACGOSAMY3	160	(+)	CGACG	S000205
CACTFTPPCA1	175	(-)	YACT	S000449
**ACGTATERD1**	**186**	**(-)**	**ACGT**	**S000415**
**ACGTATERD1**	**186**	**(+)**	**ACGT**	**S000415**
HEXAMERATH4	192	(+)	CCGTCG	S000146
CGACGOSAMY3	193	(-)	CGACG	S000205
**CRTDREHVCBF2**	**194**	**(-)**	**GTCGAC**	**S000411**
**CBFHV**	**194**	**(-)**	**RYCGAC**	**S000497**
**CRTDREHVCBF2**	**194**	**(+)**	**GTCGAC**	**S000411**
**CBFHV**	**194**	**(+)**	**RYCGAC**	**S000497**
CAREOSREP1	203	(-)	CAACTC	S000421
EECCRCAH1	203	(+)	GANTTNC	S000494
WBOXHVISO1	217	(+)	TGACT	S000442
WRKY71OS	217	(+)	TGAC	S000447
WBOXNTERF3	217	(+)	TGACY	S000457
GATABOX	235	(+)	GATA	S000039
GT1CONSENSUS	235	(+)	GRWAAW	S000198
IBOXCORE	235	(+)	GATAA	S000199
ROOTMOTIFTAPOX1	238	(-)	ATATT	S000098
ARR1AT	245	(+)	NGATT	S000454
**DOFCOREZM**	**254**	**(-)**	**AAAG**	**S000265**
**LTRE1HVBLT49**	**255**	**(-)**	**CCGAAA**	**S000250**
MYBPZM	274	(-)	CCWACC	S000179
AMMORESIIUDCRNIA1	274	(+)	GGWAGGGT	S000374
DPBFCOREDCDC3	286	(-)	ACACNNG	S000292
PREATPRODH	305	(-)	ACTCAT	S000450
CACTFTPPCA1	308	(-)	YACT	S000449
**RHERPATEXPA7**	**315**	**(+)**	**KCACGW**	**S000512**
**ABRELATERD1**	**316**	**(-)**	**ACGTG**	**S000414**
**ACGTATERD1**	**317**	**(-)**	**ACGT**	**S000415**
**ACGTATERD1**	**317**	**(+)**	**ACGT**	**S000415**
**GATABOX**	**336**	**(-)**	**GATA**	**S000039**
DOFCOREZM	357	(+)	AAAG	S000265
REALPHALGLHCB21	370	(-)	AACCAA	S000362
MYBPLANT	372	(-)	MACCWAMC	S000167
MYBPZM	372	(-)	CCWACC	S000179
BOXLCOREDCPAL	372	(-)	ACCWWCC	S000492
BS1EGCCR	383	(+)	AGCGGG	S000352
DPBFCOREDCDC3	385	(-)	ACACNNG	S000292
CACTFTPPCA1	394	(-)	YACT	S000449
GTGANTG10	410	(+)	GTGA	S000378
RAV1AAT	420	(-)	CAACA	S000314
GATABOX	444	(+)	GATA	S000039
RYREPEATLEGUMINBOX	459	(-)	CATGCAY	S000100
RYREPEATGMGY2	459	(-)	CATGCAT	S000105
RYREPEATBNNAPA	460	(-)	CATGCA	S000264
SORLIP2AT	471	(-)	GGGCC	S000483

MYBCORE	475	(+)	CNGTTR	S000176
MYB1LEPR	477	(+)	GTTAGTT	S000443
MYBPLANT	487	(+)	MACCWAMC	S000167
CANBNNAPA	490	(+)	CNAACAC	S000148
CACTFTPPCA1	499	(-)	YACT	S000449
EECCRCAH1	512	(+)	GANTTNC	S000494
REALPHALGLHCB21	519	(-)	AACCAA	S000362
MYBATRD22	520	(-)	CTAACCA	S000175
MYB1AT	520	(-)	WAACCA	S000408
MYB1LEPR	522	(+)	GTTAGTT	S000443
ABRERATCAL	536	(+)	MACGYGB	S000507
CGCGBOXAT	537	(-)	VCGCGB	S000501
CGCGBOXAT	537	(+)	VCGCGB	S000501
EECCRCAH1	543	(+)	GANTTNC	S000494
INTRONLOWER	547	(+)	TGCAGG	S000086
ACGTATERD1	577	(-)	ACGT	S000415
ACGTATERD1	577	(+)	ACGT	S000415
CACTFTPPCA1	581	(-)	YACT	S000449
CAATBOX1	589	(+)	CAAT	S000028
**NODCON2GM**	**596**	**(+)**	**CTCTT**	**S000462**
**OSE2ROOTNODULE**	**596**	**(+)**	**CTCTT**	**S000468**
**GATABOX**	**613**	**(-)**	**GATA**	**S000039**
**MYBST1**	**613**	**(-)**	**GGATA**	**S000180**
REBETALGLHCB21	613	(-)	CGGATA	S000363
**CGCGBOXAT**	**616**	**(-)**	**VCGCGB**	**S000501**
**ABRERATCAL**	**616**	**(-)**	**MACGYGB**	**S000507**
**CGCGBOXAT**	**616**	**(+)**	**VCGCGB**	**S000501**
**CURECORECR**	**624**	**(-)**	**GTAC**	**S000493**
**CURECORECR**	**624**	**(+)**	**GTAC**	**S000493**
ACGTATERD1	629	(-)	ACGT	S000415
ACGTATERD1	629	(+)	ACGT	S000415
SEF3MOTIFGM	646	(+)	AACCCA	S000115
**MYBCOREATCYCB1**	**652**	**(+)**	**AACGG**	**S000502**
DOFCOREZM	663	(-)	AAAG	S000265
AACACOREOSGLUB1	675	(+)	AACAAAC	S000353
MYB1AT	678	(+)	WAACCA	S000408
SORLIP4AT	681	(-)	GTATGATGG	S000485
**ARFAT**	**690**	**(-)**	**TGTCTC**	**S000270**
**SEBFCONSSTPR10A**	**690**	**(-)**	**YTGTCWC**	**S000391**
**SURECOREATSULTR11**	**690**	**(+)**	**GAGAC**	**S000499**
PRECONSCRHSP70A	706	(+)	SCGAYNRNNNNNNNNNNNNNNNHD	S000506
CACTFTPPCA1	731	(-)	YACT	S000449

//

>TaF3h-B2

ATTTGCAACCGAGCGAGCTTAGATGGTTAGATATTTCTTGTGGTGGAACTAGCCCATCATGGTTCATAGTCCTAAGGCCTT
ATTTGGTTTGGAGGAATTTCATAGGACTTTTATACGATAGGATTCTTATAGGAAATTTTTCTTAAAGCCTATTAATTCATA
GGAATGAATTTTTATTCCTACATAGAATTGGTTCCTATCCTTCACACTTTAAAGTAAAATAAATATGAATCTAGACTCACT
GGAAAAAAATCATATTATGTGAAACTCATAGGATTTGAGAGACTTGTCATCTCATTTCCTACAAGTTTTCTATTTCTATGA
TAATGGTATATCGTATGAACCAAAAGAAGCCTAATTTGACACTCGTGCATGCATTTTTCTGAATTTATTTCAGACTTTCTG
GTGATGTTCATTCAGTGCGAGACATCTCCCTAATTTAAAGATGTTGTGCCGGTTCAGTATTTCAAAGGTACTCAGGCGTTC
ATAGGCATCTCCCTAATTTAAATATATGTACCCTCCTTTCCATCTATAATCATACGAGACAGACGTACAGATCGATAACTG
GTAGATAGCG

```
ATTTGCAACCGAGCGAGCTTAGATGGTTAGATATTTCTTGTGGTGGAACTAGCCCATCATGGTTCATAGTCCTAAGGCCTTATTTGGTTTGGAGGAATTT   100
TAAACGTTGGCTCGCTCGAATCTACCAATCTATAAAGAACACCACCTTGATCGGGTAGTACCAAGTATCAGGATTCCGGAATAAACCAAACCTCCTTAAA

CATAGGACTTTTATACGATAGGATTCTTATAGGAAATTTTTCTTAAAGCCTATTAATTCATAGGAATGAATTTTTATTCCTACATAGAATTGGTTCCTAT   200
GTATCCTGAAAATATGCTATCCTAAGAATATCCTTTAAAAAGAATTTCGGATAATTAAGTATCCTTACTTAAAAATAAGGATGTATCTTAACCAAGGATA

CCTTCACACTTTAAAGTAAAATAAATATGAATCTAGACTCACTGGAAAAAAATCATATTATGTGAAACTCATAGGATTTGAGAGACTTGTCATCTCATTT   300
GGAAGTGTGAAATTTCATTTTATTTATACTTAGATCTGAGTGACCTTTTTTTAGTATAATACACTTTGAGTATCCTAAACTCTCTGAACAGTAGAGTAAA

CCTACAAGTTTTCTATTTCTATGATAATGGTATATCGTATGAACCAAAAGAAGCCTAATTTGACACTCGTGCATGCATTTTTCTGAATTTATTTCAGACT   400
GGATGTTCAAAAGATAAAGATACTATTACCATATAGCATACTTGGTTTTCTTCGGATTAAACTGTGAGCACGTACGTAAAAGACTTAAATAAAGTCTGA

TTCTGGTGATGTTCATTCAGTGCGAGACATCTCCCTAATTTAAAGATGTTGTGCCGGTTCAGTATTTCAAAGGTACTCAGGCGTTCATAGGCATCTCCCT   500
AAGACCACTACAAGTAAGTCACGCTCTGTAGAGGGATTAAATTTCTACAACACGGCCAAGTCATAAAGTTTCCATGAGTCCGCAAGTATCCGTAGAGGGA

AATTTAAATATATGTACCCTCCTTTCCATCTATAATCATACGAGACAGACGTACAGATCGATAACTGGTAGATAGCG                         600
TTAAATTTATATACATGGGAGGAAAGGTAGATATTAGTATGCTCTGTCTGCATGTCTAGCTATTGACCATCTATCGC
```

TF_motif_seq_0246: TF_motif_seq_0256: TF_motif_seq_0257:
TF_motif_seq_0266: TF_motif_seq_0282: TF_motif_seq_0316:
TF_motif_seq_0327: TF_motif_seq_0335: TF_motif_seq_0347:
TF_motif_seq_0386: TF_motif_seq_0422: TF_motif_seq_0508:
TF_motif_seq_0002: TF_motif_seq_0031: TF_motif_seq_0192:
TF_motif_seq_0238: TF_motif_seq_0259: TF_motif_seq_0274:
TF_motif_seq_0283: TF_motif_seq_0321: TF_motif_seq_0334:
TF_motif_seq_0350: TF_motif_seq_0372: TF_motif_seq_0380:
TF_motif_seq_0387: TF_motif_seq_0389: TF_motif_seq_0390:
TF_motif_seq_0391: TF_motif_seq_0392: TF_motif_seq_0397:
TF_motif_seq_0429:

Factor or Site Name	Loc.(Str.)	Signal Sequence	SITE #
MYBCORE	6 (-)	CNGTTR	S000176
MYBATRD22	24 (-)	CTAACCA	S000175
MYB1AT	24 (-)	WAACCA	S000408
GATABOX	30 (+)	GATA	S000039
ROOTMOTIFTAPOX1	31 (+)	ATATT	S000098
POLLEN1LELAT52	34 (-)	AGAAA	S000245
SITEIIATCYTC	51 (-)	TGGGCY	S000474
CARGATCONSENSUS	79 (-)	CCWWWWWWGG	S000404
CARGATCONSENSUS	79 (+)	CCWWWWWWGG	S000404
TATABOX5	81 (+)	TTATTT	S000203
REALPHALGLHCB21	85 (-)	AACCAA	S000362
MYB1AT	86 (-)	WAACCA	S000408
EECCRCAH1	96 (+)	GANTTNC	S000494
DOFCOREZM	109 (-)	AAAG	S000265
GATABOX	118 (+)	GATA	S000039
ARR1AT	122 (+)	NGATT	S000454
GT1CONSENSUS	133 (+)	GRWAAW	S000198
TAAAGSTKST1	146 (+)	TAAAG	S000387
DOFCOREZM	147 (+)	AAAG	S000265
TATABOX3	153 (+)	TATTAAT	S000110
INRNTPSADB	165 (-)	YTCANTYY	S000395
SEF4MOTIFGM7S	172 (+)	RTTTTTR	S000103
POLASIG1	175 (-)	AATAAA	S000080
BOXIINTPATPB	186 (+)	ATAGAA	S000296
CAATBOX1	191 (-)	CAAT	S000028
CCAATBOX1	191 (-)	CCAAT	S000030
REALPHALGLHCB21	192 (-)	AACCAA	S000362
GATABOX	200 (-)	GATA	S000039
MYBST1	200 (-)	GGATA	S000180
GTGANTG10	206 (-)	GTGA	S000378
CACTFTPPCA1	209 (+)	YACT	S000449
NTBBF1ARROLB	215 (-)	ACTTTA	S000273
TAAAGSTKST1	215 (+)	TAAAG	S000387
DOFCOREZM	216 (+)	AAAG	S000265
CACTFTPPCA1	218 (-)	YACT	S000449

TATABOX5	222	(-)	TTATTT	S000203
SEF1MOTIF	223	(-)	ATATTTAWW	S000006
POLASIG1	223	(+)	AATAAA	S000080
ROOTMOTIFTAPOX1	227	(-)	ATATT	S000098
ARR1AT	233	(-)	NGATT	S000454
GTGANTG10	242	(-)	GTGA	S000378
CACTFTPPCA1	243	(+)	YACT	S000449
PYRIMIDINEBOXHVEPB1	247	(-)	TTTTTTCC	S000298
GT1CONSENSUS	247	(+)	GRWAAW	S000198
GT1CONSENSUS	248	(+)	GRWAAW	S000198
GT1GMSCAM4	248	(+)	GAAAAA	S000453
ARR1AT	254	(-)	NGATT	S000454
ROOTMOTIFTAPOX1	258	(+)	ATATT	S000098
GTGANTG10	265	(+)	GTGA	S000378
EECCRCAH1	267	(-)	GANTTNC	S000494
PREATPRODH	270	(+)	ACTCAT	S000450
ARR1AT	277	(+)	NGATT	S000454
SURECOREATSULTR11	286	(+)	GAGAC	S000499
BIHD1OS	292	(+)	TGTCA	S000498
WRKY71OS	293	(-)	TGAC	S000447
INRNTPSADB	298	(+)	YTCANTYY	S000395
GT1CONSENSUS	301	(-)	GRWAAW	S000198
SURE1STPAT21	313	(-)	AATAGAAAA	S000186
POLLEN1LELAT52	314	(-)	AGAAA	S000245
BOXIINTPATPB	315	(-)	ATAGAA	S000296
POLLEN1LELAT52	320	(-)	AGAAA	S000245
BOXIINTPATPB	321	(-)	ATAGAA	S000296
GATABOX	327	(+)	GATA	S000039
GT1CONSENSUS	327	(+)	GRWAAW	S000198
IBOXCORE	327	(+)	GATAA	S000199
S1FBOXSORPS1L21	331	(+)	ATGGTA	S000223
**GATABOX**	**337**	**(-)**	**GATA**	**S000039**
**REALPHALGLHCB21**	**346**	**(+)**	**AACCAA**	**S000362**
DOFCOREZM	351	(+)	AAAG	S000265
WBOXATNPR1	365	(+)	TTGAC	S000390
BIHD1OS	366	(-)	TGTCA	S000498
WRKY71OS	366	(+)	TGAC	S000447
**DPBFCOREDCDC3**	**368**	**(+)**	**ACACNNG**	**S000292**
CACTFTPPCA1	369	(+)	YACT	S000449
**RHERPATEXPA7**	**372**	**(-)**	**KCACGW**	**S000512**
RYREPEATLEGUMINBOX	374	(-)	CATGCAY	S000100
RYREPEATBNNAPA	375	(-)	CATGCA	S000264
RYREPEATLEGUMINBOX	377	(+)	CATGCAY	S000100
RYREPEATGMGY2	377	(+)	CATGCAT	S000105
RYREPEATBNNAPA	377	(+)	CATGCA	S000264
GT1CONSENSUS	383	(-)	GRWAAW	S000198
GT1GMSCAM4	383	(-)	GAAAAA	S000453
POLLEN1LELAT52	385	(-)	AGAAA	S000245
POLASIG1	393	(-)	AATAAA	S000080
TATABOX5	394	(+)	TTATTT	S000203
EECCRCAH1	402	(+)	GANTTNC	S000494
DOFCOREZM	404	(-)	AAAG	S000265
POLLEN1LELAT52	405	(-)	AGAAA	S000245
GTGANTG10	411	(+)	GTGA	S000378
ARFAT	430	(-)	TGTCTC	S000270
SURECOREATSULTR11	430	(+)	GAGAC	S000499
TAAAGSTKST1	447	(+)	TAAAG	S000387
DOFCOREZM	448	(+)	AAAG	S000265

NODCON1GM	448	(+)	AAAGAT	S000461
OSE1ROOTNODULE	448	(+)	AAAGAT	S000467
RAV1AAT	453	(-)	CAACA	S000314
CACTFTPPCA1	467	(-)	YACT	S000449
**ERELEE4**	**470**	**(+)**	**AWTTCAAA**	**S000037**
DOFCOREZM	475	(+)	AAAG	S000265
**CURECORECR**	**479**	**(-)**	**GTAC**	**S000493**
**CURECORECR**	**479**	**(+)**	**GTAC**	**S000493**
CACTFTPPCA1	480	(+)	YACT	S000449
SEF1MOTIF	510	(-)	ATATTTAWW	S000006
TATABOXOSPAL	511	(-)	TATTTAA	S000400
ROOTMOTIFTAPOX1	514	(-)	ATATT	S000098
CURECORECR	521	(-)	GTAC	S000493
CURECORECR	521	(+)	GTAC	S000493
DOFCOREZM	529	(-)	AAAG	S000265
ARR1AT	541	(-)	NGATT	S000454
ARFAT	549	(-)	TGTCTC	S000270
SEBFCONSSTPR10A	549	(-)	YTGTCWC	S000391
SURECOREATSULTR11	549	(+)	GAGAC	S000499
ACGTATERD1	556	(-)	ACGT	S000415
ACGTATERD1	556	(+)	ACGT	S000415
CURECORECR	558	(-)	GTAC	S000493
CURECORECR	558	(+)	GTAC	S000493
MYBCORE	570	(-)	CNGTTR	S000176
MYB2AT	570	(+)	TAACTG	S000177
MYB2CONSENSUSAT	570	(+)	YAACKG	S000409
GATABOX	579	(+)	GATA	S000039

//
